# Unveiling the vulnerability of the human abducens nerve: insights from comparative cranial base anatomy in mammals and primates

**DOI:** 10.3389/fnana.2024.1383126

**Published:** 2024-04-29

**Authors:** Liat Rotenstreich, Ayelet Eran, Yoav Siegler, Rachel Grossman, Nir Edery, Roni Cohen, Assaf Marom

**Affiliations:** ^1^Laboratory for Anatomy and Human Evolution, The Farkas Family Center for Anatomical Research and Education, Rappaport Faculty of Medicine, Department of Neuroscience, Technion – Israel Institute of Technology, Haifa, Israel; ^2^Neuroradiology Unit, Department of Radiology, Rambam Medical Center, Haifa, Israel; ^3^Department of Obstetrics and Gynecology, Rambam Medical Center, Haifa, Israel; ^4^Department of Neurosurgery, Rambam Medical Center, Haifa, Israel; ^5^Department of Pathology, Kimron Veterinary Institute, Bet Dagan, Israel; ^6^Edmond and Lily Safra Center for Brain Sciences, Hebrew University of Jerusalem, Jerusalem, Israel

**Keywords:** abducens nerve, basicranial flexion, cranial base, Dorello canal, eye movement, internal carotid artery, lateral rectus muscle, petrosphenoidal ligament

## Abstract

The topographic anatomy of the abducens nerve has been the subject of research for more than 150 years. Although its vulnerability was initially attributed to its length, this hypothesis has largely lost prominence. Instead, attention has shifted toward its intricate anatomical relations along the cranial base. Contrary to the extensive anatomical and neurosurgical literature on abducens nerve anatomy in humans, its complex anatomy in other species has received less emphasis. The main question addressed here is why the human abducens nerve is predisposed to injury. Specifically, we aim to perform a comparative analysis of the basicranial pathway of the abducens nerve in mammals and primates. Our hypothesis links its vulnerability to cranial base flexion, particularly around the sphenooccipital synchondrosis. We examined the abducens nerve pathway in various mammals, including primates, humans (*N* = 40; 60% males; 40% females), and human fetuses (*N* = 5; 60% males; 40% females). The findings are presented at both the macroscopic and histological levels. To associate our findings with basicranial flexion, we measured the cranial base angles in the species included in this study and compared them to data in the available literature. Our findings show that the primitive state of the abducens nerve pathway follows a nearly flat (unflexed) cranial base from the pontomedullary sulcus to the superior orbital fissure. Only the gulfar segment, where the nerve passes through Dorello’s canal, demonstrates some degree of variation. We present evidence indicating that the derived state of the abducens pathway, which is most pronounced in humans from an early stage of development, is characterized by following the significantly more flexed basicranium. Overall, the present study elucidates the evolutionary basis for the vulnerability of the abducens nerve, especially within its gulfar and cavernous segments, which are situated at the main synchondroses between the anterior, middle, and posterior cranial fossae—a unique anatomical relation exclusive to the abducens nerve. The principal differences between the pathways of this nerve and those of other cranial nerves are discussed. The findings suggest that the highly flexed human cranial base plays a pivotal role in the intricate anatomical relations and resulting vulnerability of the abducens nerve.

## 1 Introduction and aims

### 1.1 Historical background

More than 150 years have passed since the pioneering descriptions by Grüber, Dorello, and Gradenigo of the involvement of the abducens nerve in skull base lesions and its possible neuroanatomical underpinnings (Grüber, 1859; Dorello, 1905; Gradenigo, 1907; Ambekar et al., 2012; Kshettry et al., 2013; Reddy et al., 2016). To date, the neurological, neuroanatomical, and neurosurgical literature continues to grapple with attempts to uncover the reasons for the notable susceptibility of this nerve in various clinical contexts (Hanson et al., 2004; Baidoo and Tubbs, 2015). Whether focusing on its length, relation to adjacent structures, or suggesting a vascular mechanism for its susceptibility, attempts to explain the vulnerability of the abducens nerve have centered on its uniquely tortuous course through the cranial base. These attempts have resulted in the methodological division of the course of the abducens nerve into several segments, each characterized by specific anatomical relations (Iaconetta et al., 2007). In particular, several authors have focused on the intricate relations of the abducens nerve in the gulfar (petroclival) segment, where it passes from the posterior to the middle cranial fossa (Umansky et al., 1991, 1992; Tubbs et al., 2012), a narrow region of intersection between the sphenoid bone, the petrous part of the temporal bone, and the clivus of the occipital bone. In Primo Dorello’s words, *“Dato lo spazio limitato in cui decorre il nervo abducens in corrispondenza dell’apice della rocca, non è difficile comprendere come per modificazioni di questo spazio esso possa andare sogetto non difficilmente a compressione”* (Dorello, 1905; p. 216). Namely, “Given the limited space in which the abducens nerve runs at the apex of the rock [i.e., petrous bone], it is not difficult to understand how, due to modifications of this space, it can easily be subjected to compression.” Dorello attempted to explain the mechanism of injury to the abducens nerve within a specific pathological condition, i.e., inflammation of the middle ear cavity within the petrous bone. According to Dorello, in this case, the abducens nerve is compressed against the wall of the canal through which it is transmitted from the posterior to the middle cranial fossa; this canal is known today as Dorello’s canal (Umansky et al., 1991; Destrieux et al., 1997). Other authors have suggested additional mechanisms of injury, which will be summarized in section 1.3. Regardless of the mechanism of injury to the abducens nerve, in the present study, we sought to explain why the abducens nerve is prone to such injury within its course via a comparative neuroanatomical approach.

### 1.2 Anatomical framework

The human abducens nerve is one of three cranial nerves that mediate ocular motility by carrying general somatic motor axons from the brainstem to the extraocular muscles; the other two nerves are the oculomotor nerve and the trochlear nerve (Rhoton, 2000; Kiernan and Rajakumar, 2014; Kandel et al., 2021; Standring, 2021). The abducens nerve exclusively supplies the ipsilateral lateral rectus muscle (Kandel et al., 2021; Standring, 2021), which abducts the eyeball—hence, the name “abducens” (Sömmering, 1778; Porras-Gallo et al., 2019). The axons of the abducens nerve emerge from its nucleus within the tegmentum of the pons, where it is engulfed by the internal genu of facial nerve axons (Kiernan and Rajakumar, 2014). Controlled by the paramedian pontine reticular formation (PPRF), which functions as a lateral gaze brainstem center, general somatic efferent neurons of the abducens nucleus send their axons ventrally to exit the brainstem as the most medial nerve emerging through the pontomedullary sulcus (Kiernan and Rajakumar, 2014; Standring, 2021), as a single nerve trunk in more than 92% of cases (Nathan et al., 1974; Iaconetta et al., 2007).

The entire basicranial pathway of the abducens nerve has been divided into five segments according to the main anatomical relations of the nerve to its surroundings (Iaconetta et al., 2007). The cisternal segment extends from the point where the abducens nerve exits at the level of the pontomedullary sulcus to the clival dura (Iaconetta et al., 2007; Joo et al., 2012). Lying within the prepontine cistern, in this segment, the abducens nerve is supplied by branches of the basilar artery, and an important relation in this region is to the anterior inferior cerebellar artery, which typically passes below the nerve (Tubbs and Loukas, 2016; Standring, 2021). The gulfar segment begins at the point where the abducens nerve penetrates the clival dura mater, acquires a dural sleeve and enters the venous confluence situated at the junction of the clivus with the sphenoid and petrous bones (Iaconetta et al., 2007). Importantly, the dural entrance of the abducens nerve is one of the vertices of the inferomedial paraclival triangle. This triangle provides a roadmap to the surgical anatomy of this segment of the abducens nerve (Wysiadecki et al., 2021a). Within this venous gulf, the abducens nerve passes through Dorello’s canal, below the petrosphenoidal ligament of Grüber (Rhoton, 2002; Iaconetta et al., 2003; Reddy et al., 2016). Within the canal, the abducens nerve is enveloped by a dural sleeve that is attached to the ligament and to the periosteum through fibrous trabeculations (Nathan et al., 1974; Umansky et al., 1992). Importantly, the dural sleeve of the abducens nerve is separated from that of the trigeminal nerve through Meckel’s cave (Rootman et al., 2022a). The dorsal meningeal artery, a branch of the meningohypophyseal trunk, is situated medial to the abducens nerve within the canal (Liu et al., 2009). Next, the abducens nerve bends sharply over the crest of the petrosal apex and enters the cavernous sinus. In the cavernous segment, the abducens nerve runs along the inferolateral wall of the internal carotid artery and then medial and parallel to the filaments of the ophthalmic division of the trigeminal nerve (Weninger and Pramhas, 2000; Iaconetta et al., 2007; Standring, 2021; Rootman et al., 2022b). Recently, a new subdivision of the cavernous segment was proposed, based on the topographical relations to the internal carotid artery (carotid portion) and internal cavernous plexus (prefissural portion) (Wysiadecki et al., 2021b). The fissural segment of the abducens nerve is situated within the superior orbital fissure (Rootman et al., 2022c). The common tendinous ring of Zinn, a fibrous ring that surrounds the optic canal and part of the superior orbital fissure, transmits the abducens nerve into the orbital cavity along with the superior and inferior divisions of the oculomotor and nasociliary nerves (Standring, 2021; Rootman et al., 2022d). Last, the intraconal segment of the abducens nerve lies within the orbital cavity. In this segment, the abducens nerve curves laterally and branches into several fasciculi that penetrate the medial surface of the lateral rectus muscle (Nam et al., 2017; Standring, 2021).

### 1.3 Mechanisms of abducens nerve injury

In addition to the methodical segmentation of the basicranial pathway of the abducens nerve, the neuroanatomical and neurosurgical literature calls attention to two key concepts regarding the clinical anatomy of its course. First, several authors have highlighted the angulations of the course of the abducens nerve through the skull base as a reason for predisposition to injury under specific pathological conditions (Iaconetta et al., 2001, 2003, 2007). The marked bending of the abducens nerve at the level of the petrous apex, at an angle of approximately 90°, is particularly emphasized in explaining its vulnerability (Wolff, 1928; Arias, 1985; Umansky et al., 1991, 1992; Tekdemir et al., 1996; Ozveren et al., 2002a; Ozer et al., 2010; Joo et al., 2012; Azarmina and Azarmina, 2013). Other points of bending along the abducens pathway are also mentioned, e.g., the angulation between the cisternal part and the point where the abducens nerve first becomes extradural, and lateral bend in its course as it leaves Dorello’s canal and enters the cavernous sinus (Iaconetta et al., 2001). Additionally, Wysiadecki et al. (2021b) have highlighted the angulation of the nerve at the interface with the intracavernous segment of the internal carotid artery. At this juncture, the nerve consistently adheres to the posterior genu of the internal carotid artery. Second, fixation points of the abducens nerve within its segments are also discussed as a plausible mechanism of injury. In particular, rigid tethering through fibrous trabeculations within Dorello’s canal has been hypothesized to restrict its mobility under pathological conditions that cause a caudal shift of the brainstem (Umansky et al., 1992; Tsitsopoulos et al., 1996; Ozveren et al., 2002b, 2007; Liu et al., 2009; Icke et al., 2010; Tubbs et al., 2012).

The notable vulnerability of the abducens nerve has been the focus of intense microanatomical and microsurgical research. As described by Wolff (1928), “the weakling of the cranial contents, the sixth nerve may be affected in almost any type of cerebral lesion. It is thus notorious, if involved alone, for having no localizing value.” According to traditional teaching in neurology, the susceptibility of the abducens nerve is attributed to its long intracranial course (Sachsenweger, 1969; Arias, 1985; Umansky et al., 1992; Ten Donkelaar, 2011; Joo et al., 2012; Standring, 2021). However, the trochlear nerve, which exits through the dorsal aspect of the midbrain and thus has a longer course through the cranial base, is rarely injured in patients with increased intracranial pressure (Joo et al., 2012). Indeed, as demonstrated by direct measurements of the length of these cranial nerves by Hanson et al. (2004), the trochlear nerve has a longer intracranial course than the abducens nerve. Furthermore, Hanson et al. (2004) suggested that the fibrous tissues within Dorello’s canal rostrally tether the abducens nerve such that during transtentorial herniation, it is stretched and may consequently become necrotic as it enters the canal. In light of these findings, it has become evident that the vulnerability of the abducens nerve is more likely to result from its local anatomical relations through the basicranium rather than from its length.

In this regard, several specific pathophysiological mechanisms have been proposed for the notable vulnerability of the abducens nerve. First, there are a multitude of clinical reports of abducens nerve palsy secondary to direct mechanical pressure. This pathophysiological mechanism may result from vascular compression (Sarwar, 1977; Coppeto and Chan, 1982; Ohtsuka et al., 1996; Giray et al., 2005; De Ridder and Menovsky, 2007), mass effects due to space-occupying lesions (Collier, 1904; Zielinski, 1959; Hashimoto and Ohtsuka, 1998; Ziyal et al., 2006; Ayberk et al., 2008) or direct compression by the petrosphenoidal ligament (Schneider and Johnson, 1971; Tubbs et al., 2014). Second, several authors have reported injury to the abducens nerve due to ischemia or infarction resulting from vascular strangulation (Cushing, 1910; Sunderland, 1948; Lang, 1981; Donaldson and Rosenberg, 1988). Third, the stretching effect resulting from caudal displacement of the brainstem (e.g., due to herniation) on the abducens nerve has been suggested by several authors as a possible pathophysiological mechanism. In this clinical setting, the abducens nerve may become necrotic as it enters the gulfar segment due to its rostral fixation by the osteofibrous elements of Dorello’s canal (Umansky et al., 1991; Hanson et al., 2004). This pathophysiological mechanism may also underlie abducens nerve palsy in the clinical setting of decreased intracranial pressure (Bryce-Smith and Macintosh, 1951; Insel et al., 1980; Espinosa et al., 1993; Follens et al., 2001; Niedermüller et al., 2002; Arcand et al., 2004; Azarmina and Azarmina, 2013; Hofer and Scavone, 2015), trauma (Schneider and Johnson, 1971; Takagi et al., 1976; Uzan et al., 1996; Ozveren et al., 2001; Sam et al., 2004), or space-occupying lesions (Collier, 1904).

### 1.4 Comparative anatomy of the abducens nerve

There is a large body of literature on the pathophysiology of abducens nerve vulnerability, and it suggests that the susceptibility of this nerve to injury in various clinical contexts results from its unique pathway through the cranial base and its distinct anatomical relations to other structures rather than its length. Importantly, the available literature regarding the basicranial pathway of the abducens nerve in mammals and primates is very limited. Such descriptions of the abducens nerve in veterinary texts are often general and do not detail the specific anatomical relations of the nerve through the basicranium (Popesko, 1961; Adams, 2004; Dyce et al., 2009; Budras et al., 2010; Marom, 2010; Evans and de Lahunta, 2012; König and Liebich, 2014; Evans and de Lahunta, 2016). In addition, and significantly, there is limited information available concerning the vulnerability of the abducens nerve in nonhuman species. According to the existing data, in contrast to humans, in nonhuman species, the abducens nerve is seldom compromised in clinical scenarios involving alterations in intracranial pressure or trauma. Furthermore, when affected, it tends to be affected in conjunction with the oculomotor nerve rather than in isolation (Penderis, 2003; Platt and Olby, 2014). Similarly, information regarding cranial base flexion, as expressed by the angles between its components, is primarily available for humans and primates (Ross and Ravosa, 1993; Ross and Henneberg, 1995; Lieberman et al., 2000; Lieberman, 2011), with substantially less data available for other mammalian species (De Beer, 1937; Lieberman et al., 2008).

### 1.5 Aims of the present study

The present study does not seek to propose an additional mechanism of injury but rather to explain why the abducens nerve is predisposed to involvement in these clinical situations in the first place. Here, we aim to perform a comparative analysis of the basicranial pathway of the abducens nerve in mammals and primates to explain the uniqueness of the pathway of the abducens nerve, which possibly underpins its notable vulnerability.

Our working hypothesis is that the abducens nerve pathway in humans is unique and that its vulnerability may result from two possible factors. First, the increased vulnerability of the abducens nerve may be caused by its anatomical relations within the Dorello canal (Ekanem et al., 2023; Plutecki et al., 2023). A second possible factor is that the abducens nerve pathway is exceptionally close to the midline compared to other cranial nerves. Specifically, we hypothesize that due to this proximity, angulations in its course are brought about by basicranial flexion, an evolutionary process that has become fully fledged in humans (Lieberman et al., 2000; Lieberman, 2011).

## 2 Materials and methods

### 2.1 Ethical statement

The cadaveric head specimens of nonhuman species used in the present study were procured from several sources during the years 2021–2023. These include the Pre-Clinical Research Authority at the Rappaport Faculty of Medicine (Technion – Israel Institute of Technology, Israel); the Zoological Center Ramat Gan (Israel); the Edmund and Lily Safra Center for Brain Sciences (The Hebrew University of Jerusalem); the Kimron Veterinary Institute (Ministry of Agriculture, Bet Dagan, Israel); and the Steinhardt Museum of Natural History (Tel-Aviv University). Since these specimens were acquired postmortem, Institutional Animal Care and Use Committee (IACUC) approval was not needed.

The human samples in the present study included samples from adults and fetuses. The adult human samples were obtained from the Farkas Family Center for Anatomical Research and Education at the Technion (Israel Institute of Technology) following the prescribed institutional ethical regulations. In this part of the study, we examined 40 adult hemicranial bases obtained from formaldehyde-fixed body donors with a mean age of 71 years (60% males; 40% females). The human fetal samples consisted of 5 spontaneously aborted human fetuses (60% males; 40% females) obtained from the Department of Obstetrics and Gynecology at the Rambam Health Care Campus in Haifa (Israel), in compliance with Institutional Ethical Approval no. RMB-0538-22.

### 2.2 Samples

In the present study, we investigated the cranial base pathway of the abducens nerve along the cranial base of various mammals. The relations of the abducens nerve to adjacent structures along its pathway are systematically described at both the gross anatomical and microanatomical levels within the cisternal, gulfar, cavernous, fissural, and intraconal segments of its course in the following mammalian orders: Artiodactyla, Carnivora, Chiroptera, Diprotodontia, Eulipotyphla, Hyracoidea, Lagomorpha, Peramelemorpia, Perissodactyla, Primates and Rodentia. Notably, our sample also included 10 hemicranial bases of human fetuses, with gestational ages ranging from 14.6 weeks to 21.3 weeks. Table 1 provides a comprehensive summary of the species examined within each order and the corresponding number of specimens included in our study.

**Table 1:** Species examined in the present study and sample size.

**Species**	**Common name**	**Gross anatomy**	**Histology**
Artiodactyla *Ovis aries* (2) *Sus scrofa* (2)	Domestic sheep Wild boar	+ +	- -
Carnivora *Canis lupus familiaris* (3) *Felis silvestris catus* (2) *Vormela peregusna* (1)	Domestic dog Domestic cat Marbled polecat	+ + -	- - -
Chiroptera *Rossettus aegyptiacus* (2) *Eptesicus serotinus* (1)	Fruit bat Serotine bat	- -	+ -
Diprotodontia *Bettongia gaimardi* (1) *Macropus gigantus* (1) *Macropus rufogriseus* (1) *Trichosurus vulpecula* (1) *Vombatus ursinus* (1)	Eastern bettong Eastern gray kangaroo Red necked wallaby Common brushtail possum Common wombat	- - - - -	- - - - -
Eulipotyphla *Erinaceus concolor* (1)	Hedgehog	-	-
Hyracoidea *Procavia capensis* (1)	Rock hyrax	-	-
Lagomorpha *Lepus capensis* (2)	Cape hare	-	+
Peramelemorpia *Isoodon macrourus* (1)	Bandicoot	-	-
Perissodactyla *Equus quagga* (1)	Zebra	+	-
Primates *Callithrix* sp. (1) *Cebus* sp. (1) *Cercopithecus mitis* (1) *Chlorocebus pygerythrus* (1) *Gorilla gorilla* (5) *Macaca* sp.(1) *Pan troglodytes* (1) *Saguinus* sp. (1) *Saimiri sciureus* (2) *Homo sapiens* (45)	Marmoset Capuchin monkey Diademed monkey Vervet monkey Western lowland gorilla Rhesus macaque Chimpanzee Tamarin Common squirrel monkey Human	+ - - - + + + - + +	- - - - - - - - + +
Rodents *Mus musculus* (5) *Rattus rattus* (5)	House mouse Black rat	+ +	+ +

### 2.3 Gross anatomy

To prepare the specimens for examination, the heads were carefully severed at the level of the C_1_ vertebra and subsequently immersed in a 4% formaldehyde solution for fixation for a minimum duration of 4 weeks before they were dissected. Gross anatomical dissection of the cranial base and the abducens nerve pathway was performed following the methods applied by Joo et al. (2012).

First, we performed a macroscopic evaluation of the abducens nerve pathway through traditional dissection methods. Small specimens (e.g., mouse and rat head specimens) were dissected using a Nikon SMZ 25 stereomicroscope and a Zeiss TIVATO 700 surgical microscope. We applied a retrograde approach to identify the abducens nerve and its course, i.e., the nerve was first identified within the orbital cavity through its penetration of the medial surface of the lateral rectus muscle and then followed posteriorly to the point where it traverses the meninges (Iaconetta et al., 2007; Joo et al., 2012; Nam et al., 2017; Standring, 2021). The steps included in this approach are described below.

For each cadaveric head specimen, a midline incision was made along the scalp, extending from the external occipital protuberance to the supraorbital ridge. The scalp was then reflected, exposing the calvaria and the temporalis muscle. The latter was dissected out of the temporal fossa, allowing full exposure of the superior and lateral surfaces of the cranium. This step is particularly important for facilitating the identification of the lateral rectus muscle in mammalian species, where the orbital cavity is not separated from the temporal fossa by a bony septum. Subsequently, craniotomy was performed using an electric autopsy bone saw, and the brain was carefully extracted, with special attention given to preservation of the cranial nerves along the basicranium. Then, access into the orbital cavity was gained via the orbital plate of the frontal bone anteriorly and the lesser wing of the sphenoid bone posteriorly. The orbital periosteum and adipose tissue were removed, allowing adequate exposure and identification of the extraocular muscles, nerves, and blood vessels. The superior orbital fissure was opened to identify the oculomotor, trochlear, and abducens nerves, as well as the ophthalmic division of the trigeminal nerve, as they traverse the common tendinous ring (annulus of Zinn) and enter the orbital cavity. Along the lateral border of the orbit, the lateral rectus muscle was exposed and identified in all specimens, with the abducens nerve bundles penetrating its medial surface (Nam et al., 2017; Standring, 2021). Subsequently, a retrograde approach was applied to trace the abducens nerve pathway from the orbital cavity (intraconal segment), through the fissural segment, into the cavernous and gulfar segments, and finally into the cisternal part. Several anatomical resources were used to accurately identify anatomical and neuroanatomical structures (Sicher and DuBrul, 1970; Standring, 2021; Rootman et al., 2022a,b,c,d). Our findings were documented using a Canon EOS 90D camera.

### 2.4 Histology

For histological analyses, preparations of the parasellar region including the abducens nerve pathway were resected from the available specimens (see Table 1). The dimensions of samples harvested for histological processing were determined according to cranial size. For smaller specimens, e.g., those from mice, rats and bats, we used the entire cranial base. For larger specimens, e.g., rabbit, squirrel monkey and human specimens, specific anatomical boundaries were defined for harvesting full-thickness samples. The anterior border was marked by the crista galli of the ethmoid bone, the posterior border by the basion (most anterior point on the rim of the foramen magnum), and the lateral border by a sagittal plane lateral to the petrous apex. The samples were carefully collected *en bloc* using a bone saw and preserved in a 4% formaldehyde solution. To prepare the samples for histological analysis, the initial stage of a decalcification procedure was employed; the samples were immersed in MoL-Decalcifier Milestone solution, with a pH range of 7.2 to 7.4, at 37°C for varying durations ranging from 24 h to 49 days, depending on the size of the specimen. In the present study, we utilized a manual (physical) testing through probing or bending to detect hardness of the specimen. This method was employed to ascertain the completion of sample decalcification, following the approach outlined by Suvarna et al. (2018). Following decalcification, the samples underwent standard histological tissue processing, adjusted to their dimensions (Kiernan, 2015). Each specimen was embedded in paraffin and sectioned at a thickness of 5 μm. Different section planes, mostly parasagittal, were considered for each specimen to demonstrate the abducens pathway along the cranial base. Sections were stained using Masson’s trichrome, which enables clear differentiation between nerve, muscle, bone, and connective tissue. This method was selected because the different segments of the abducens nerve pathway include various anatomical elements.

### 2.5 Cranial base angle measurement

Cranial base angulation refers to the relations among the cranial base planes resulting from flexion and extension of the ethmoid, sphenoid, and basioccipital bones on the sagittal plane (Lieberman and McCarthy, 1999). The most common measure, termed cranial base angle 1 (CBA1), quantifies the angle between two lines on the midsagittal plane: one connects the anterior margin of the foramen magnum (basion) with the center of the hypophyseal fossa, and the other is from the sella turcica to the foramen cecum (Ross and Ravosa, 1993; Lieberman and McCarthy, 1999; Lieberman et al., 2000). Thus, CBA1 is a measure of the angle between the prechordal and parachordal portions of the cranial base (Lieberman, 2011; Schoenwolf et al., 2014).

To study the variations in the degree of cranial base flexion among mammalian species, specimens included in this study were scanned using either a μCT or a CT scanner, depending on specimen size. Larger specimens were scanned using a Siemens CT scanner through a dual-energy protocol at a slice thickness of 1 mm in the Department of Radiology, Rambam Health Care Campus in Haifa (Israel). For small specimens, μCT scanning was applied using a SkyScan 1,276 desktop scanner (Bruker, Kontich, Belgium). The scan parameters included the following: filter, Al Cu; voltage, 100 kV; current, 40 μA; rotation step, 0.5 through 360 degrees; 2-frame averaging; and total resolution, 42 μm. All the resulting projection images were reconstructed using NRecon software (v.1.7.4.5, Bruker, Kontich, Belgium) with post-alignment and beam-hardening corrections. 3D analysis of the scans was performed using Amira-Avizo software (Version 2021.1) (Stalling et al., 2005). This process included manual and threshold-based segmentation. The segmented images were subjected to surface reconstruction using the ‘generate surface’ module of the software, followed by clipping on the midsagittal plane (Ito, 2019). Landmarks were positioned at the basion, the center of the sella turcica, and the foramen cecum, which together define the cranial base angle CBA1 (Lieberman and McCarthy, 1999). Importantly, certain mammalian species, primarily rodents, do not possess a clearly defined hypophyseal fossa. In these species, a landmark was placed at the midsphenoidal synchondrosis instead of at the center of the sella turcica, aligning with the methodology outlined by Lieberman et al. (2008). The distribution of CBA1 values between two groups: primates and nonprimates were compared using a two-sample t test to compare the means of two independent samples with a significance level of alpha = 0.05.

## 3 Results

**Figure 1:**
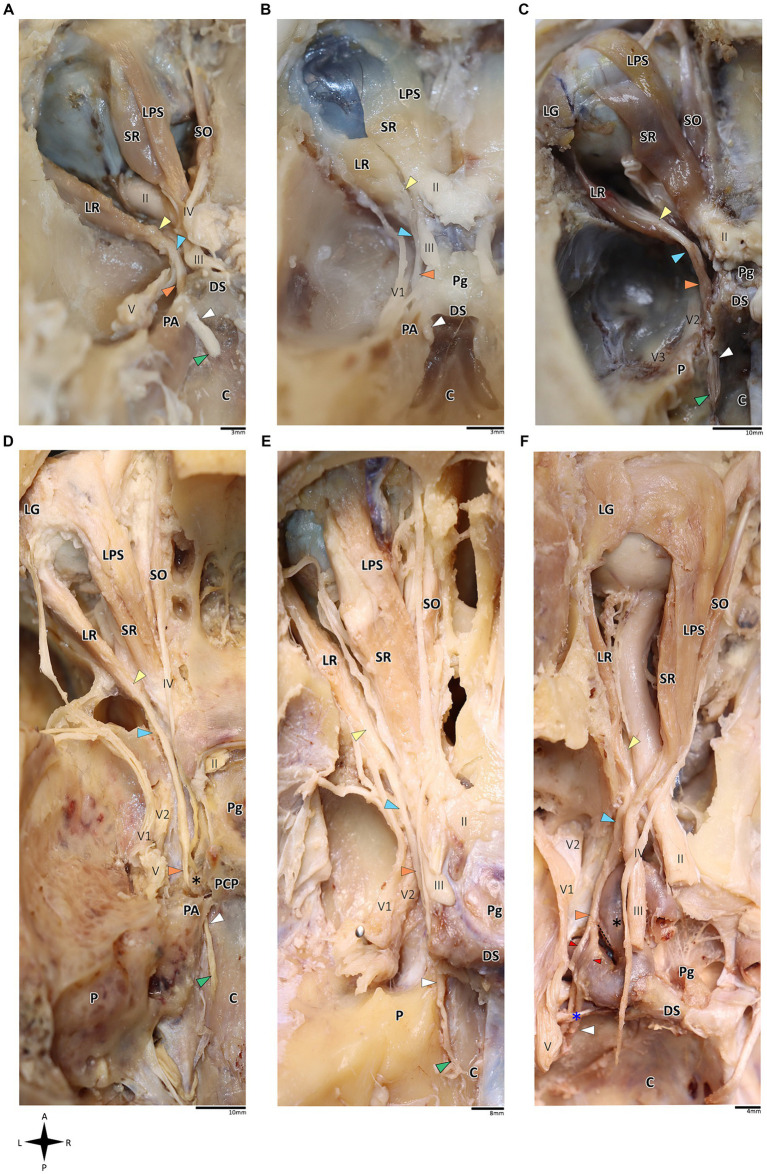
Anatomical dissection of the abducens nerve in humans and primates. Gross anatomical dissection of the basicranium and orbital cavity in the primate order, featuring *Saimiri sciureus* **(A)**, *Callithrix* sp. **(B)**, *Macaca* sp. **(C)**, *Gorilla gorilla* **(D)**, *Pan troglodytes* **(E)**, and *Homo sapiens* **(F)**, demonstrating the course of the abducens nerve pathway along the cranial base from the cisternal to the intraconal segment. Arrows indicate the abducens nerve segments as follows: cisternal segment (green); gulfar segment (white); cavernous segment (orange); fissural segment (blue); intraconal segment (yellow). In panel **F**, red arrow indicates pseudobranching of the abducens nerve, and dashed line indicates communication between the abducens nerve and carotid sympathetic plexus. C, clivus; DS, dorsum sella; LG, lacrimal gland; LPS, levator palpebrae superioris muscle; LR, lateral rectus muscle; P, petrous bone; Pg, pituitary gland; SO, superior oblique muscle; SR, superior rectus muscle; II, optic nerve; III, oculomotor nerve; IV, trochlear nerve; V, trigeminal nerve; V1, ophthalmic nerve; V2, maxillary nerve. The petrosphenoidal ligament is marked by a blue asterisk, and the internal carotid artery by a black asterisk. A, anterior; L, left; P, posterior; R, right.

**Figure 2:**
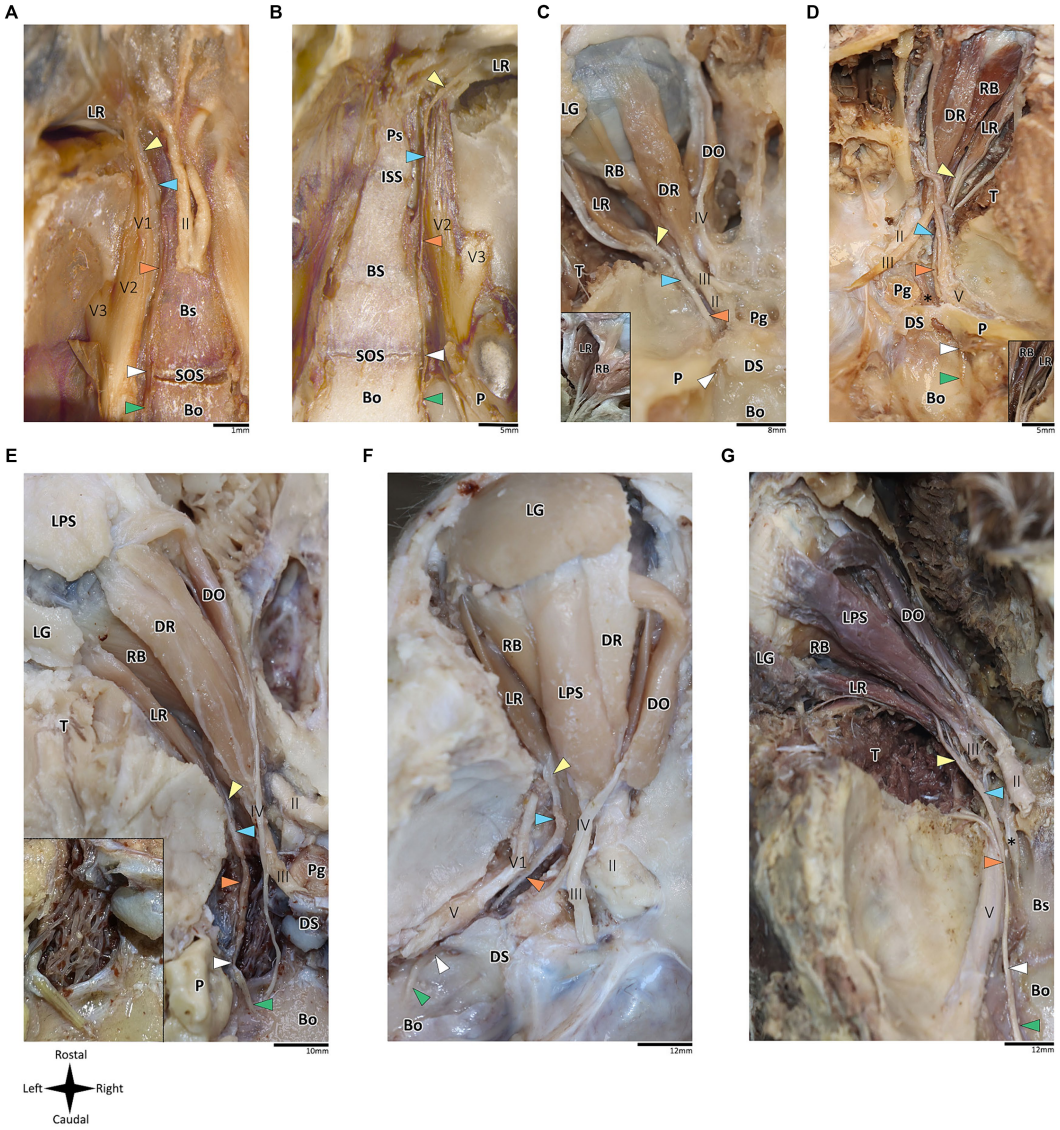
Anatomical dissection of the abducens nerve in mammals. Gross anatomical dissection of the orbital cavity and basicranium in *Mus musculus* **(A)**, *Rattus rattus* **(B)**, *Felis silvestris catus* **(C)**, *Canis lupus familiaris* **(D)**, *Sus scrofa* **(E)**, *Ovis aries* **(F)** and *Equus quagga* **(G)**, demonstrating the course of the abducens nerve pathway along the cranial base from the cisternal to the intraconal segment. Abducens nerve segments are indicated by arrow heads as follows: cisternal segment (green); gulfar segment (white); cavernous segment (orange); fissural segment (blue); intraconal segment (yellow). In panels C and D, smaller box shows branching of nerve bundles from the abducens nerve to the retractor bulbi muscle. In panel E, smaller box shows an enlarges image of the carotid rete. Bo, basioccipital bone; Bs, basisphenoid bone; DO, dorsal oblique muscle; DR, Dorsal rectus muscle; DS, Dorsum sella; ISS, intersphenoidal synchondrosis; LG, lacrimal gland; LPS, levator palpebrae superioris muscle; LR, lateral rectus muscle; P, petrous bone; Pg, Pituitary gland; Ps, presphenoid bone; RB, retractor bulbi muscle; SOS, sphenooccipital synchondrosis; T, temporalis muscle; II, optic nerve; III, Oculomotor nerve; IV, trochlear nerve; V, trigeminal nerve; V1, ophthalmic nerve; V2, maxillary nerve; V3, mandibular nerve; The internal carotid artery is labeled by an asterisk.

**Figure 3:**
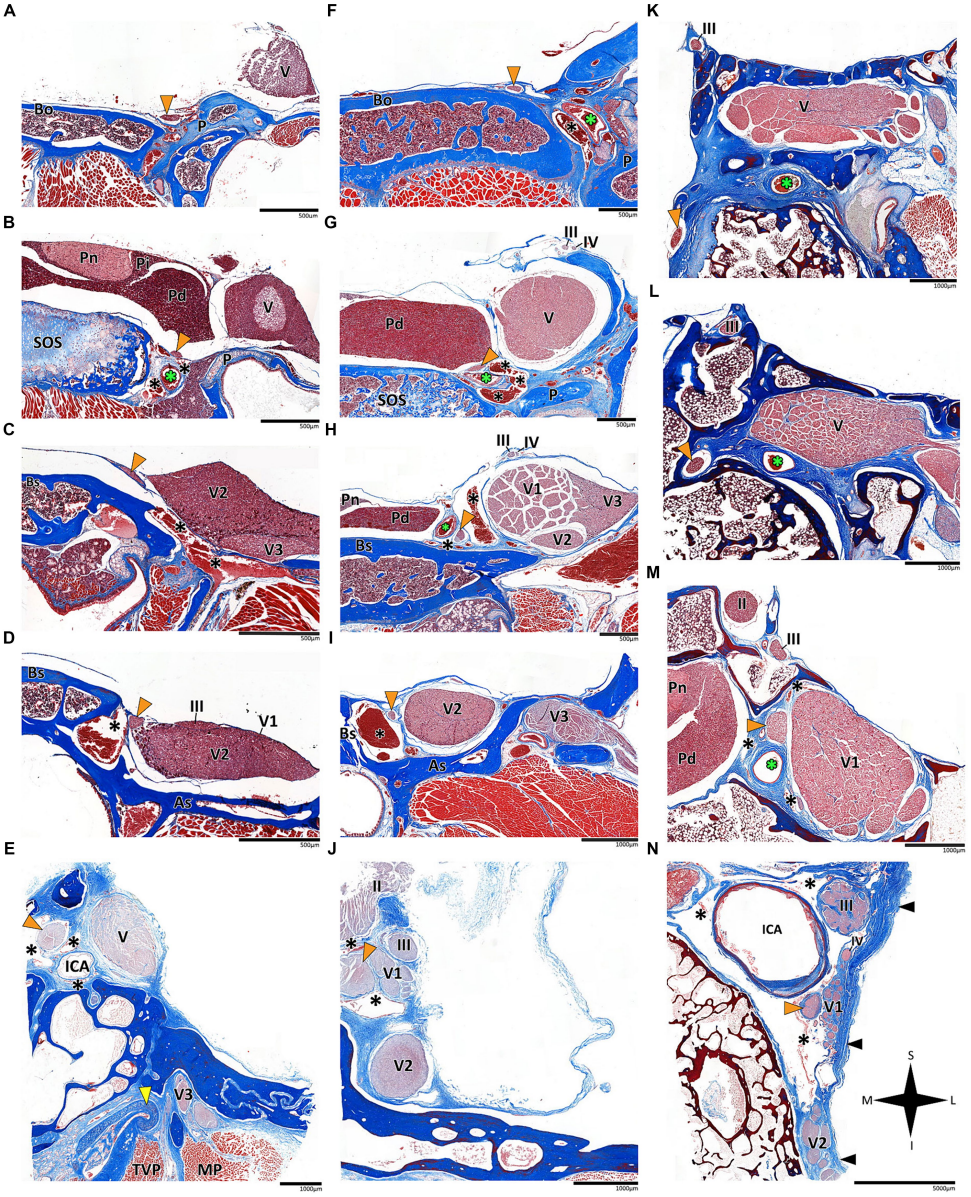
Histological coronal sections of the abducens nerve pathway. Histological coronal sections representing the abducens nerve’s basicranial pathway in the following mammalian species: *Mus musculus* **(A–D)**, *Rattus rattus* **(F–I)**, *Lepus capensis* **(K–M)**, *Saimiri sciureus* **(E,J)** and *Homo sapiens* **(N)**. For each species, panels are arranged in a posterior to anterior sequence, following segments of the abducens nerve: the cisternal segment (panels **A,F,K**); the gulfar segment (panels **B,G**); upon entering the cavernous segment (panels **C,E,H,L**); and in the cavernous sinus (panels **D,I,J,M,N**). As, Alisphenoid bone; Bo, basioccipital bone; Bs, basisphenoid bone; ICA, internal carotid artery; MP, Medial pterygoid muscle; P, petrous bone; Pd, pars distalis of pituitary gland (adenohypophysis); Pi, pars intermedia of pituitary gland; Pn, pars nervosa of pituitary gland (neurohypophysis); SOS, sphenooccipital synchondrosis; TVP, Tensor veli palatini muscle; yellow arrowhead, eustachian tube cartilage; black arrowhead, lateral dural wall of cavernous sinus. III, oculomotor nerve; IV, trochlear nerve; V, trigeminal nerve; V1, ophthalmic nerve; V2, maxillary nerve; V3, mandibular nerve. The abducens nerve is consistently highlighted across all sections by an orange arrowhead, the internal carotid artery by a green asterisk, and the cavernous sinus by a black asterisk. I, inferior; L, lateral; M, medial; S, superior.

**Figure 4:**
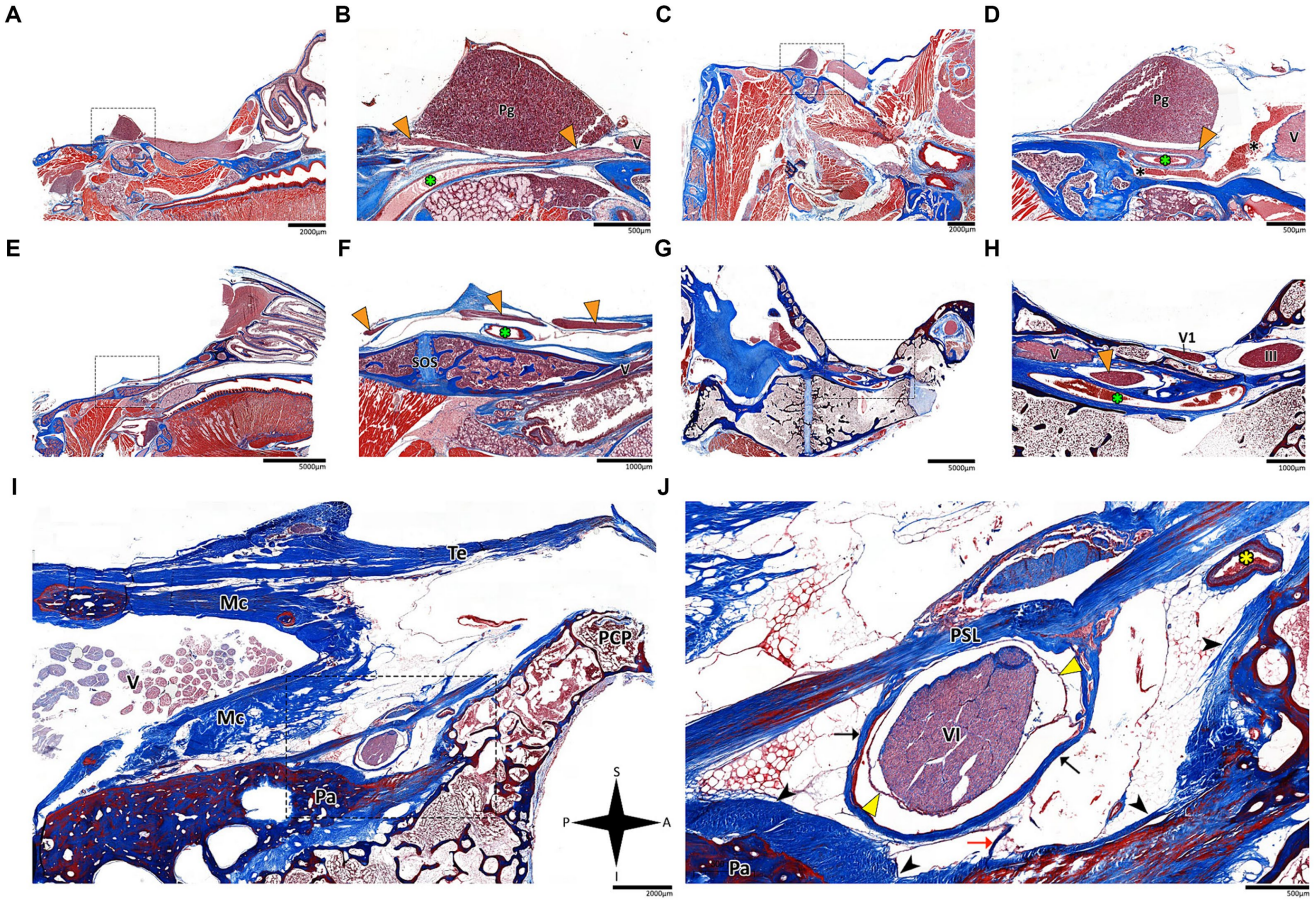
Histological sagittal sections of the abducens nerve pathway. Sagittal sections of the abducens nerve’s pathway along the cranial base in several species: *Mus musculus* **(A,B)**; *Rattus rattus* **(C,D)**; *Rossettus aegyptiacus* **(E,F)**; *Lepus capensis* **(G,H)**; and *Homo sapiens* **(I,J)**. In each of these couples, the right image focuses on the area outlined by a dashed rectangle on the left image. The upper row of panels captures the gulfar segment of the abducens nerve. The middle row captures the cavernous segment of the abducens nerve. The lower demonstrates the passage of the abducens nerve through Dorello’s canal in adult humans. Note the spatial relations of Meckel’s cave to Dorello’s canal and the tentorium. Mc, Meckel’s cave; Pa, petrous apex; PCP, posterior clinoid process; Pg, pituitary gland; PSL, petrosphenoidal ligament; SOS, sphenooccipital synchondrosis; Te, tentorium cerebelli; III, oculomotor nerve; V, trigeminal nerve; V1, ophthalmic nerve; VI, abducens nerve. Yellow arrowhead, arachnoid membrane; Black arrowhead, periosteal dura; Orange arrowhead, abducens nerve; Black arrow, dural sleeve; Red arrow, fibrous trabeculations; Black asterisk, cavernous sinus; Green asterisk, internal carotid artery; Yellow asterisk, dorsal meningeal artery. A, anterior; I, inferior; P, posterior; S, superior.

To investigate the course of the abducens nerve along the cranial base from a comparative viewpoint, we conducted a comprehensive series of dissections of the orbital cavity and basicranium of species classified in five mammalian orders (see Table 1). Our gross anatomical observations were confirmed and enhanced by a series of histological investigations. In addition, we measured the CBA1 in each of these specimens. The gross anatomical dissection findings are presented for humans and primates in Figure 1 and for other mammals in Figure 2. The results of the histological analyses are presented in Figure 3 (coronal sections) and Figure 4 (sagittal sections).

### 3.1 Human and primate samples

On dissection of the human and primate orbital cavity, across all the examined species, we consistently identified seven extraocular muscles. These include the four rectus muscles, i.e., the superior rectus muscle, inferior rectus muscle, medial rectus muscle and lateral rectus muscle; two oblique muscles, i.e., the superior oblique muscle and the inferior oblique muscle; and the levator palpebrae superioris muscle. The attachment points of these muscles and their innervation patterns align with previous descriptions (Yamashita et al., 1980; Shimokawa et al., 2002; Spencer and Porter, 2006; König and Liebich, 2014; Bohlen et al., 2019; Standring, 2021). Particular attention was directed toward the lateral rectus muscle since it is supplied by the abducens nerve. This muscle originated consistently from the lateral aspect of the annulus of Zinn and the orbital surface of the greater wing of the sphenoid bone. Its course followed a remarkably similar route across all the primate species, proceeding forward along the lateral aspect of the orbit and ultimately inserting into the lateral surface of the sclera, with the abducens nerve penetrating its medial (ocular) surface (Nam et al., 2017; Standring, 2021). Subsequently, we methodically traced the abducens nerve in a retrograde fashion along its five accepted segments in humans, as postulated by Iaconetta et al. (2007).

### 3.2 Mammalian samples

In addition to the orbital muscles described for humans and primates, in the mammalian species possessing a nictitating membrane, we identified a variable number of bellies of the retractor bulbi muscle, which is consistently situated closer to the globe than the other extraocular muscles. The lateral rectus muscle was identified in all the mammalian species included in this study. Our findings revealed a markedly similar anatomical pattern in the course of the abducens nerve across mammals, with the gulfar segment demonstrating the most variable pattern compared to other segments. While our dissection was retrograde, findings are reported for each segment along the cranial base from the cisternal to the intraconal segment.

### 3.3 Cisternal segment

In the human and primate cranial base, this segment of the abducens nerve is entirely confined to the posterior cranial fossa. It extends from the point where the nerve emerges from the pontomedullary sulcus to the point where it pierces the dura and becomes situated between the dura mater and the clival periosteum, as the nerve straddles the petrous bone. Within this segment, the abducens nerve is accompanied by an arachnoid sleeve (see, for example, Figures 3F, 4F). Initially, it is very close to the midline and then immediately ascends superiorly, anteriorly, and laterally along the clivus of the occipital bone until it pierces the dura mater medial and adjacent to the petrous apex. In all the mammalian specimens we examined the nerve maintains a similar course, traveling straight and forward along the lateral margin of the basilar part of the basioccipital and basisphenoid bones. Notably, in the human sample and in the macaque specimen we noted an anatomical variant of this segment of the abducens nerve, emerging as a conglomeration of rootlets (see Figure 1C).

### 3.4 Gulfar segment

In the human cranial base, the gulfar segment transmits the abducens nerve from the posterior cranial fossa, where it is entirely intradural, into the middle cranial fossa, where it acquires a dural sleeve. This envelope continues to accompany the nerve as it enters a venous sinus gulf, formed at the intersection between the sphenoid bone, petrous apex, and clivus (see Figures 3B,G–I, 4F,H,J). This venous confluence is formed by the cavernous sinus, vertebral basilar venous plexus, and superior petrosal sinus and additionally communicates with the inferior petrosal sinus laterally and inferiorly. In the human configuration of this segment, the abducens nerve relates to two important bony elements; it passes medial to the petrous apex of the temporal bone and lateral to the dorsum sella and the posterior clinoid process of the sphenoid bone. Importantly, within the gulf, the nerve passes through Dorello’s canal. This space is bounded superiorly by the petrosphenoidal ligament of Grüber, medially by the lateral edge of the dorsum sella, laterally by the petrosal apex at the sphenopetrosal synchondrosis, and inferiorly by the surface of the clivus (Iaconetta et al., 2003; Reddy et al., 2016) (see Figures 1F, 4I,J). Within this segment, remnants of arachnoid were detected under the dural covering (see Figures 4I,J). In addition, the dorsal meningeal artery was detected within the proximal part of the Dorello canal, medial to the abducens nerve (see Figures 4I,J) as part of the contents of the inferomedial paraclival triangle (McCormack et al., 2021; Wysiadecki et al., 2021a).

Our findings in nonhuman primates align with the results from our previous research on these species (Marom, 2010). Similar to humans, the canal is formed between the petrous apex and the posterior clinoid process, and its roof may be formed by the fusion of these elements. However, while in humans the roof of Dorello’s canal is often formed by Grüber’s ligament, which may be ossified or unossified (Kshettry et al., 2013; Tubbs et al., 2014), such a ligament was not identified in any of the primates examined here. In the Gorilla, Callithrix and Saimiri specimens, after the abducens nerve enters the gulfar segment, it passes through Dorello’s canal, bounded superiorly by complete fusion of the petrous apex and posterior clinoid process. In chimpanzees and macaques, the petrous apex and clinoid process were not entirely fused, and the nerve followed a course through a narrow gap created between them (see Figure 1). In summary, the gulfar segment in primates exhibits a wide range of variation.

According to our observations in mammals, the gulfar segment exhibits the most variable anatomical pattern in this group. In rodents, while the basicranium features a petrous apex, a distinct sella turcica, i.e., the hypophyseal fossa, is absent. The pituitary gland was identified in all the specimens above the flat surface of the sphenooccipital synchondrosis, with its posterior border lacking a developed dorsum sella and a posterior clinoid process (see Figures 2A,B). Consequently, we did not identify a gulfar segment of the abducens nerve in rodents. Instead, at the level of the sphenooccipital synchondrosis, the nerve passes under the lateral part of the gland and superior to the internal carotid artery to reach the cavernous segment. These observations were also confirmed by histological analyses (see Figures 3B,G, 4A–D).

Regarding Artiodactyla, our observations in *Sus scrofa* revealed that the abducens nerve passes through the osseous concavity of the petroclival region within a large venous confluence but is situated in close proximity to the petrous apex rather than the dorsum sella. In other words, it occupies the most lateral position within the venous gulf. Additionally, it passes inferior to the trigeminal ganglion. In contrast, in *Ovis aries*, the nerve travels adjacent to the posterior clinoid process, i.e., it occupies the most medial position within the venous gulf. Importantly, in both species, we identified both the dorsum sella and the petrous apex, with a relatively large space between them. However, they were not linked by a ligament, such that we could not define Dorello’s canal within the gulfar segment in these species. Our findings in Perissodactyla were similar to those observed in *Sus scrofa* (see Figures 2E–G).

In Carnivora, there is a space between the dorsum sella and the petrous apex, bridged by an ossification of the dura mater. In *Canis lupus familiaris*, the abducens nerve passes from the posterior cranial fossa into the middle cranial fossa inferior to the petrous part of the temporal bone, relating to the petrooccipital canal (Evans and De Lahunta, 2012). The anatomical configuration in *Felis catus* is similar, as the abducens nerve was identified within a canal formed between the petrous apex laterally and the posterior clinoid process medially. This canal is homologous to Dorello’s canal in humans and functions in transmitting the nerve from the posterior to the middle cranial fossa (see Figures 2C,D).

### 3.5 Cavernous segment

In humans, the cavernous segment typically begins as the abducens nerve exits from Dorello’s canal at the level of the posterior genu of the internal carotid artery. After traversing the petroclival concavity, the nerve sharply bends into the cavernous sinus. In this region the nerve adheres to the internal carotid artery, and we observed pseudobranching of the nerve in 7 cases (17.5%; *N* = 40) (see Figure 1F). In most cases we observed a typical single trunk variant (Wysiadecki et al., 2021b). Within the sinus, the nerve runs along the inferolateral wall of the internal carotid artery, positioned medial to the ophthalmic nerve, which is embedded within the lateral dural wall of the sinus (see Figures 1F, 3N). Both the oculomotor and trochlear nerves are also embedded in the lateral wall of this sinus; however, they are situated higher in relation to the abducens nerve and to the ophthalmic nerve (see Figure 3N).

In the primates we examined, the relation of the abducens nerve to the cavernous sinus is similar to that in humans. Also similar to the human condition, the abducens nerve lies lateral to the intracavernous internal carotid artery. In particular, in the chimpanzee and the gorilla specimens we examined, we noted that the proximal portion of the cavernous segment of the abducens nerve is a location of angulation, caused by the posterior genu of the internal carotid artery. The cranial nerves embedded in the lateral wall of the sinus are the oculomotor, trochlear, ophthalmic, and maxillary nerves; the abducens nerve is more medial and therefore bears a closer relation to the internal carotid artery and the sinus (Figures 3E,J,N).

In mammals, our dissections revealed a similar anatomical pattern of this segment across all examined species. In rodents, the abducens nerve passes directly beneath the pituitary gland at the level of the sphenooccipital synchondrosis. It courses anteriorly, adjacent to the cavernous sinus, but does not enter the sinus. It travels along the lateral margin of the basisphenoid and presphenoid bones, initially gaining a position superior to the sinus. More anteriorly, it gains a position lateral to the sinus (see Figures 3A–D, 3F–I, 4A–D). Importantly, coronal sectioning revealed that the sinus has a crescent shape, with its concavity facing medially. This concavity houses the abducens nerve and the internal carotid artery. Coronal sections revealed more details regarding the anatomical relations of the abducens nerve to the internal carotid artery. In the region of the trigeminal ganglion, the nerve lies above the artery (Figures 3B,G). More distally, as the main divisions of the trigeminal nerve are identified, the abducens nerve lies inferolateral to the internal carotid artery (Figures 3D,H).

While in rodents, the main relating structure of the abducens nerve is the cavernous sinus, in *Lepus capensis* (Lagomorpha), our histological sections demonstrated a relatively modest cavernous sinus, and it is primarily surrounded by thick connective tissue. We used coronal sections to demonstrate the abducens nerve’s relation to the internal carotid artery. In the region of the trigeminal ganglion, the nerve lies medial to the artery (Figure 3L). More distally, the abducens nerve lies superior to the internal carotid artery (Figure 3M). These anatomical relations were also observed in sagittal sections of the cranial base in rodents (Figures 4A–D), lagomorpha (Figures 4G,H), and chiroptera (Figures 4E,F).

Importantly, in artiodactyla, the carotid system forms a specialized subdural meshwork of freely anastomosing arteries. This system, known as the carotid rete mirabile, replaces the internal carotid artery in supplying the cerebral arterial circle (Daniel et al., 1953; O’Brien, 2020). In the pig and sheep specimens we examined, the carotid rete is housed within the cavernous venous sinus (Figures 2E,F), and the abducens nerve was observed traversing through the arterial meshwork.

### 3.6 Fissural segment

In the human and primate cranial base, the fissural segment begins as the abducens nerve leaves the cavernous sinus and passes through the superior orbital (sphenoidal) fissure, an opening between the greater and lesser wings of the sphenoid that facilitates communication between the middle cranial fossa and the orbital cavity. Importantly, the abducens nerve is situated within the lateral margin of the central portion of the superior orbital fissure (Sicher and DuBrul, 1970; Iaconetta et al., 2007; Standring, 2021). In our dissections of the primate specimens, in the superior orbital fissure, the abducens nerve is situated medial and inferior to the frontal nerve, and lateral to the superior and inferior divisions of the oculomotor nerve.

In mammals, the course of the abducens nerve within this segment is highly preserved across all the species we examined. Within the fissure, the abducens nerve is typically situated between the frontal and oculomotor nerves. It then courses within the annulus of Zinn to enter the orbital cavity and gains a more lateral position to reach the muscle it supplies. In rodents, artiodactyls and perissodactyls, the fissural segment of the abducens nerve passes medial to the ophthalmic nerve. Our histological sagittal sections confirmed these relations also in chiroptera (Figure 4E). Importantly, in these species, the maxillary nerve (V_2_) also passes through the sphenoidal fissure, inferior to the abducens nerve (see blue arrow in the panels of Figure 2).

### 3.7 Intraconal segment

The intraconal segment constitutes the portion of the abducens nerve extending beyond the annulus of Zinn, proceeding forward along the lateral aspect of the orbital cavity. In humans, primates, and the mammalian species included in this study, we observed a consistent course of the abducens nerve within this segment. The nerve passes forward into the orbital cavity and branches into several filaments that penetrate the medial surface of the lateral rectus muscle, most frequently in its posterior one-third (Nam et al., 2017; Standring, 2021). In addition, in mammals possessing a retractor bulbi muscle, the nerve further gives off one or two smaller branches that curve medially, providing innervation to the lateral part of the retractor bulbi muscle (see Figures 2C,D).

### 3.8 Analysis of human embryos

**Figure 5:**
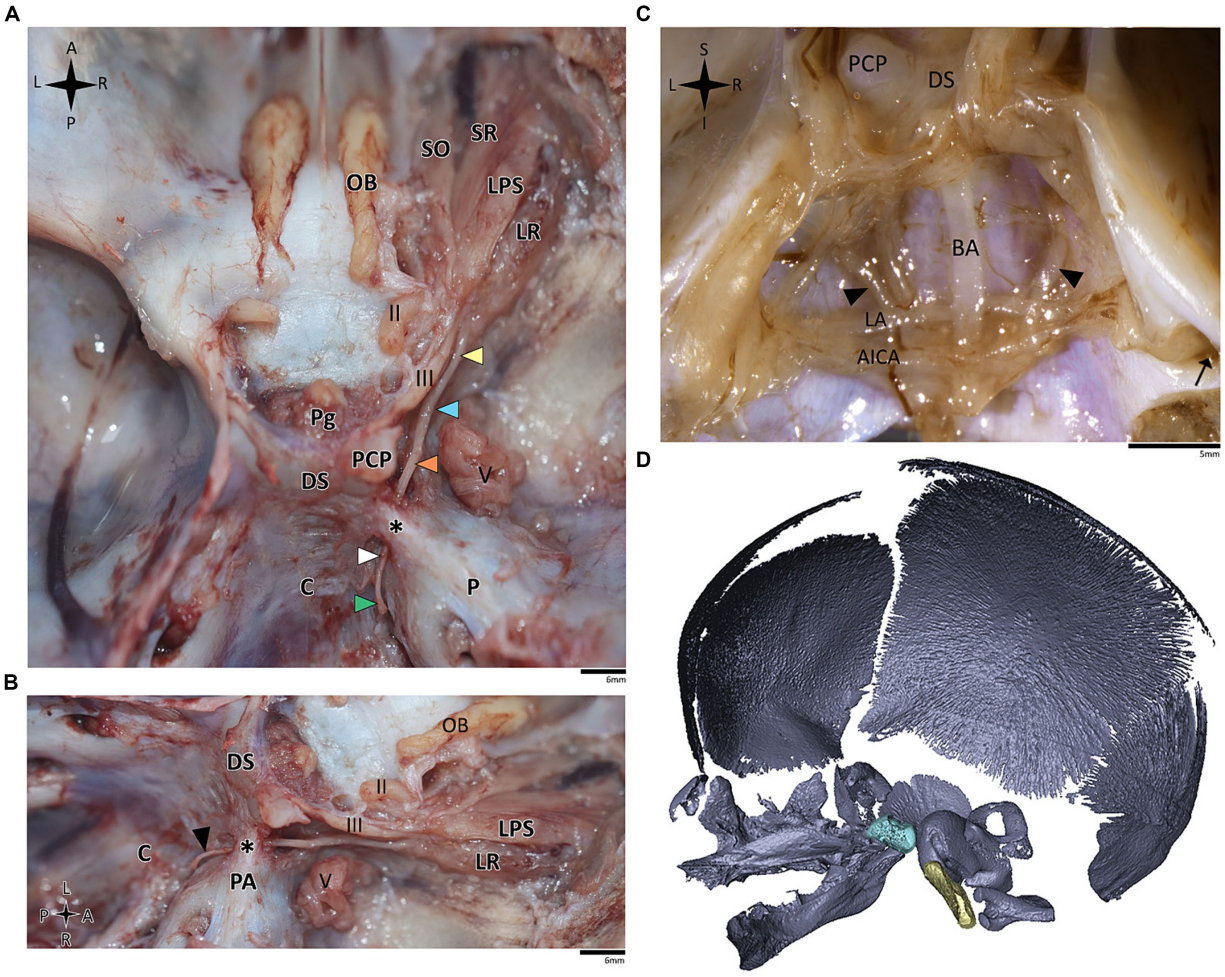
Anatomical dissection of the abducens nerve in human fetal specimens. Endocranial views of the human fetal cranial base, focusing on the abducens nerve. **(A)** superior and **(B)** lateral view of the fetal cranial base (gestational age 21 + 3). Arrowheads depict the segments of the abducens nerve segments as detailed in figure. The petrosphenoidal ligament is labeled by an asterisk. **(C)** posterior view of the clivus (gestational age 20 + 1). The brainstem was removed while retaining the arachnoid close to the cranial base, i.e., the anatomical structures are viewed here from within the subarachnoid space. Black arrowheads indicate the abducens nerve. The internal acoustic meatus is indicated by a black arrow. **(D)** 3D reconstruction of a fetal skull (gestational age 21 + 3), based on microCT scanning. The sella turcica is highlighted in pale blue, and the clivus in yellow. AICA, anterior inferior cerebellar artery; BA, basilar artery; C, clivus; DS, dorsum sella; LA, labyrinthine artery; LPS, levator palpebrae superioris muscle; LR, lateral rectus muscle; Ob, olfactory bulb; P, petrous bone; PA, petrous apex; PCP, posterior clinoid process; Pg, pituitary gland; SO, superior oblique; SR, superior rectus; II, optic nerve; III, oculomotor nerve; V, trigeminal nerve. A, anterior; I, inferior; L, left; P, posterior; R, right; S, superior.

**Figure 6:**
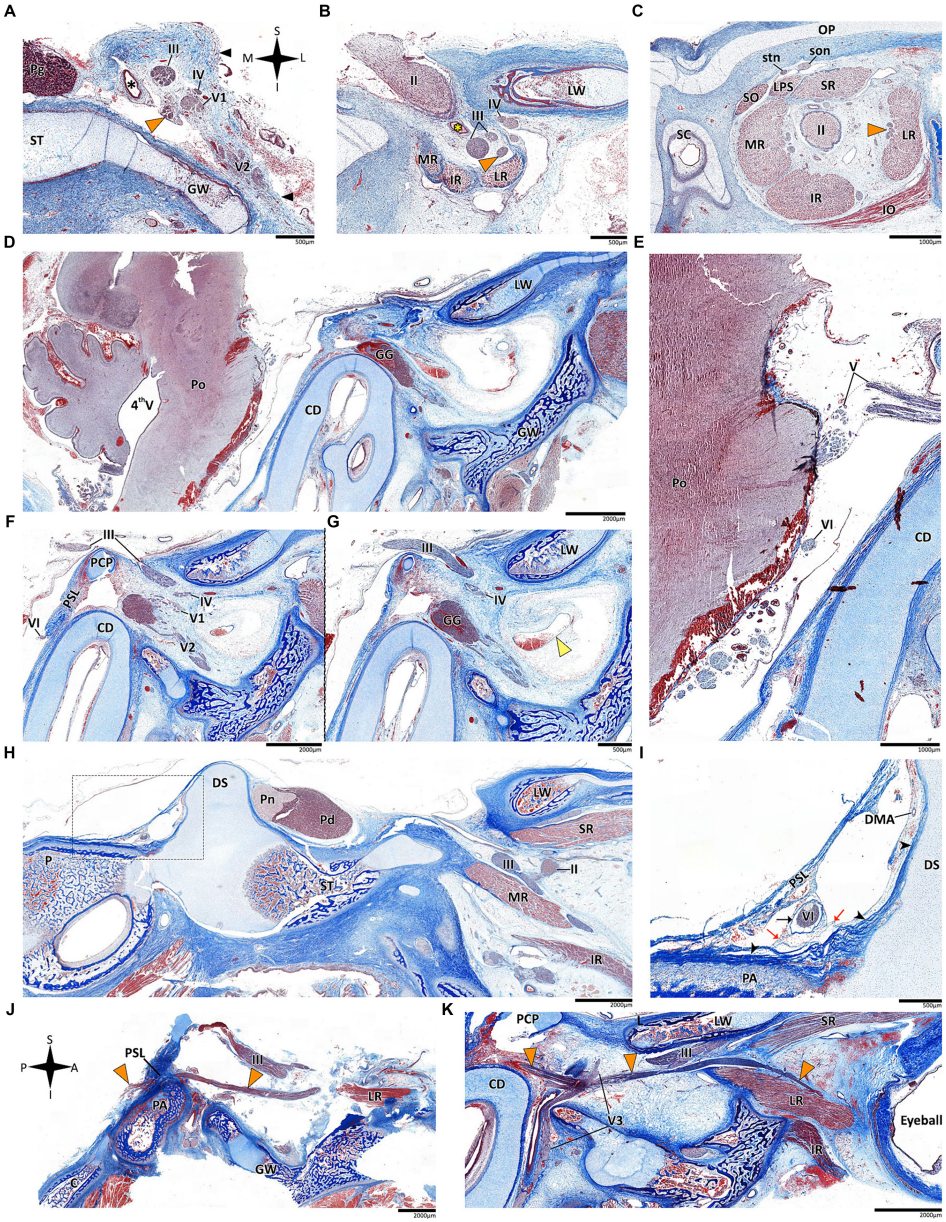
Histological sections of the abducens nerve in human fetal specimens. Panels **A–C** represent coronal sections, arranged in a posterior-to-anterior sequence, demonstrating the cavernous, fissural and intraconal segments of the abducens nerve, respectively. The abducens nerve is labeled by an orange arrowhead, and the cavernous sinus by a yellow arrowhead. The internal carotid artery is marked by a black asterisk, and the ophthalmic artery by a yellow asterisk. **(D)** sagittal section of the fetal cranial base including derivatives of the hindbrain, i.e., the pons, medulla oblongata, and cerebellum. **(E)** An enlarged image of the brainstem and cisternal segments of several cranial nerves. **(F,G)** are consecutive sagittal sections through the lateral wall of the cavernous sinus. **(H,I)** sagittal sections through Dorello’s canal. Note that **(I)** represents an enlarged view of the area marked by a dashed rectangle in panel **(H)**. **(J)** The abducens nerve is demonstrated passing from the posterior to the middle cranial fossa in a sagittal section. **(K)** The fissural and intraconal segments in a sagittal section. C, clivus; CD, cochlear duct; DMA, dorsal meningeal artery; DS, dorsum sella; GG, Gasserian Ganglion; GW, greater wing of sphenoid bone; IO, inferior oblique muscle; IR, inferior rectus muscle; LPS, levator palpebrae superioris muscle; LR, lateral rectus muscle; LW, lesser wing of sphenoid bone; MR, medial rectus muscle; OP, orbital plate of frontal bone; P, petrous bone; PA, petrous apex; PCP, posterior clinoid process; Pd, pars distalis of pituitary gland (adenohypophysis); Pg, pituitary gland; Pn, pars nervosa of pituitary gland (neurohypophysis); Po, pons; PSL, petrosphenoidal ligament; SC, superior nasal concha; SO, superior oblique muscle; son, supraorbital nerve; ST, sella turcica; stn, supratrochlear nerve; II, optic nerve; III, oculomotor nerve; IV, trochlear nerve; V1, ophthalmic nerve; V2, maxillary nerve; V3, mandibular nerve; VI, abducens nerve.

The results of our dissections of human embryonic specimens are summarized in Figure 5. Our gross anatomical observations were also confirmed by histological analyses, as summarized in Figure 6. Anatomical dissection of the embryonic cranial bases revealed a pattern similar to that of the adult configuration regarding all segments of the abducens nerve. The segments of the abducens nerve, their spatial relations, and the marked angulation within the gulfar segment appear to be acquired at a very early stage of embryonic development. In particular, the passage of the abducens nerve through the Dorello canal and the presence of the petrosphenoidal ligament were confirmed and demonstrated in all the specimens included in this study. In the analysis of fetal specimens, the dorsal meningeal artery was identified as the most medial structure within the Dorello canal (see Figure 6I).

**Figure 7:**
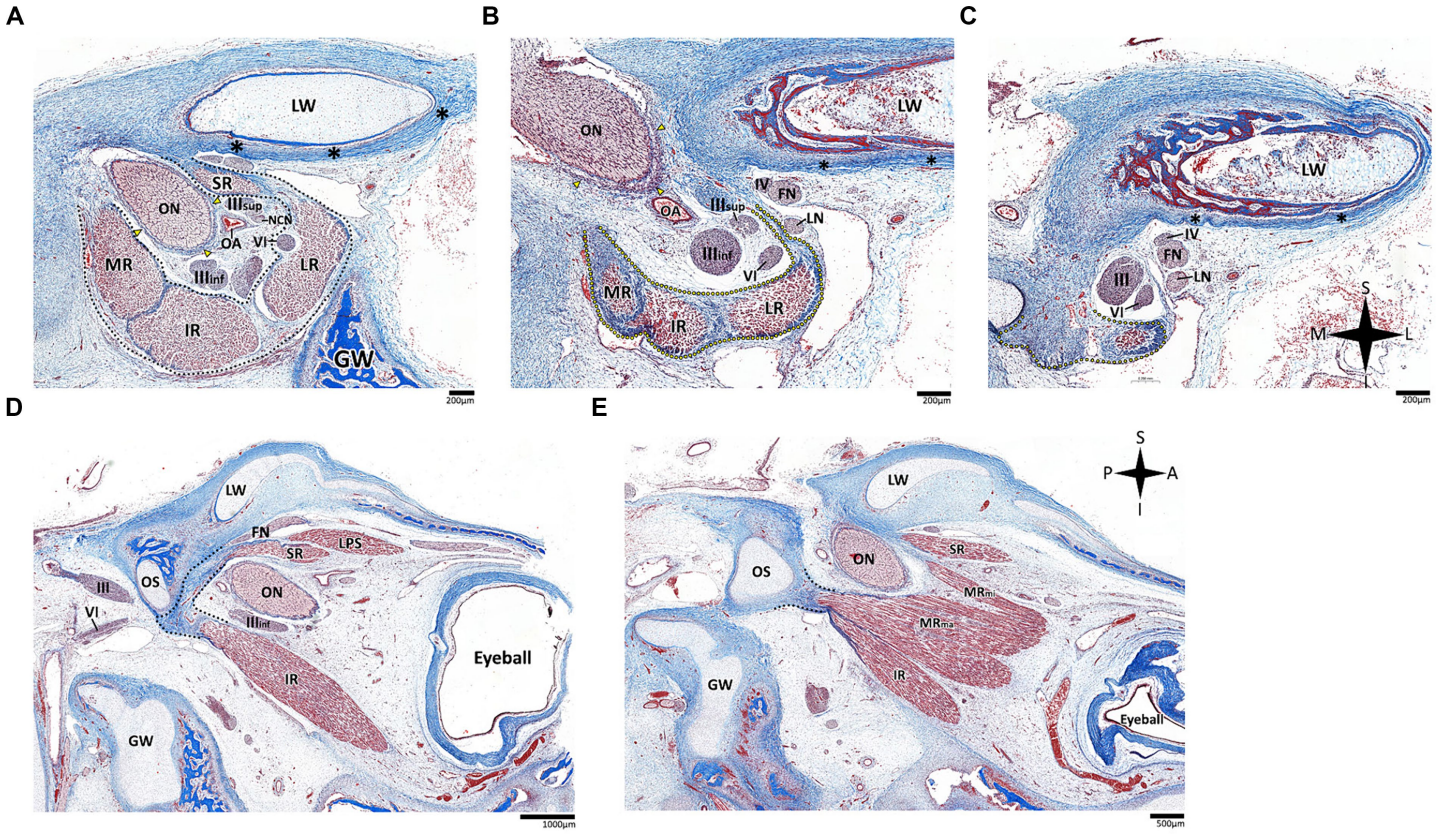
Histological sections of the fissural and intracontal segments of human fetal specimens. Panels **A–C** demonstrate a series of coronal sections through the intraconal and fissural segments of the abducens nerve in a human fetal specimen (**A** is most anterior and **C** is most posterior). The common tendinous ring is labeled by a black dotted line, connecting to the periosteal dura (black asterisk) and to the dural sheath of the optic nerve (yellow arrows). Note the common tendinous origin for the medial rectus, lateral rectus, and inferior rectus (yellow dotted line in panels **B**,**C**). Panels **D,E** demonstrate sagittal sections through the intraconal and fissural segments of the abducens nerve in a human fetal specimen (**D** is more lateral than **E**). Note the connection of the common tendinous ring (black dotted line) to the optic strut (OS). FN, frontal nerve; GW, greater wing of sphenoid bone; IR, inferior rectus muscle; LN, lacrimal nerve; LPS, levator palpebrae superioris muscle; LR, lateral rectus muscle; LW, lesser wing of sphenoid bone; MR, medial rectus muscle; MRma, major head of medial rectus muscle; MRmi, minor head of medial rectus muscle; NCN, nasociliary nerve; OA, ophthalmic artery; ON, optic nerve; SR, superior rectus muscle; IIIinf, inferior division of oculomotor nerve; IIIsup, superior division of oculomotor nerve; IV, trochlear nerve; VI, abducens nerve. A, anterior; I, inferior; L, lateral; M, medial; P, posterior; S, superior.

Histological analysis of the fissural and intraconal segments of the abducens nerve yielded several noteworthy observations regarding the relations of the common tendinous ring of Zinn. First, we noted that the superior part of the common tendinous ring is attached to the optic sheath and to the periosteum of the sphenoid bone (see Figure 7A). Next, we observed a common tendinous origin for the medial rectus, lateral rectus, and inferior rectus muscles that merges with the periosteum of the sphenoid lesser wing. The oculomotor and abducens nerve were situated superior to this common tendon (see Figures 7B,C). In addition, we noted that the annulus attaches posteriorly to the developing optic strut (see Figures 7D,E). These observations support new data on the topographical anatomy of the common tendinous ring in the adult human orbit (Kanehira et al., 2022; Lacey et al., 2022; Ma et al., 2022).

Within the range of gestational age of human embryonic specimens included in the present study, the bony components of the basicranium, i.e., the prechordal, hypophyseal, and parachordal cartilaginous plates, are still unossified. Accordingly, we could not reliably estimate the cranial base angle, as depicted in Figure 5D.

### 3.9 Cranial base angle measurement

**Figure 8:**
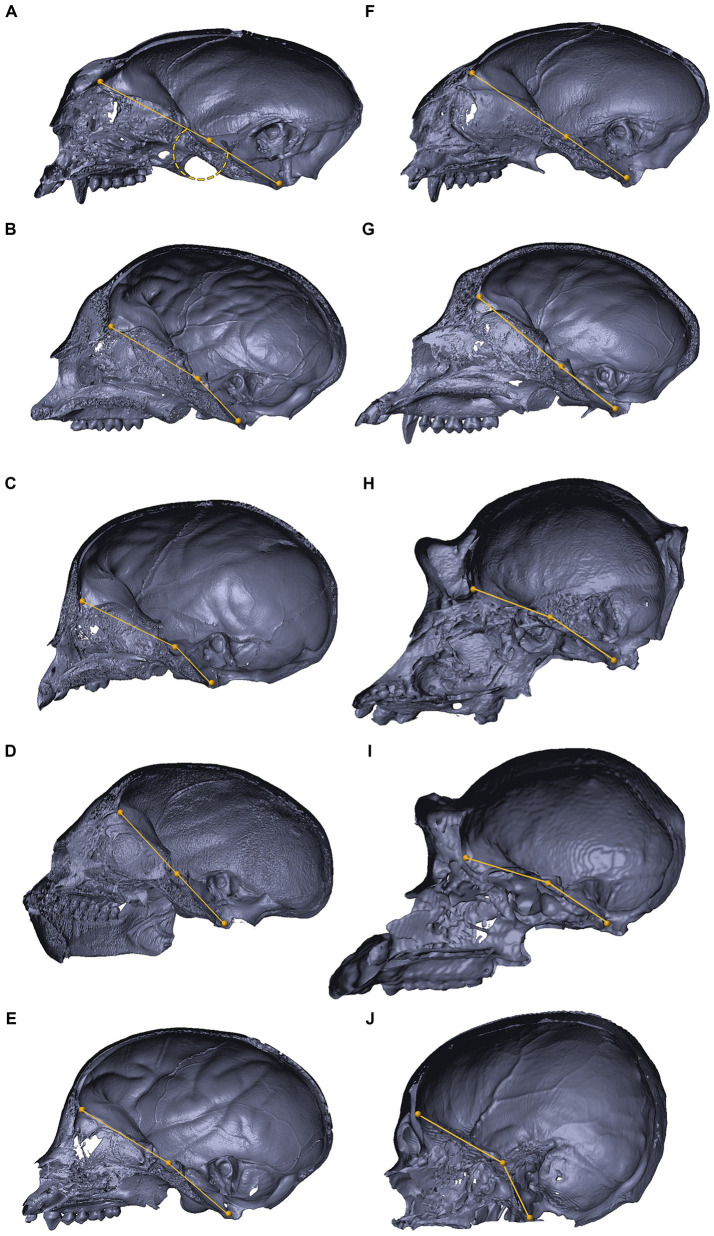
3D reconstructions of the cranial base in primates. 3D reconstructions of primate crania sectioned along the midsagittal plane, representing variations in the degree of cranial base flexion (CBA1). *Saguinus* sp. **(A)**, *Cholocebus pygerythrus* **(B)**, *Macaca fascicularis* **(C)**, *Saimiri* sp. **(D)**, *Cebus* sp. **(E)**, *Callithrix* sp. (panel **F**), *Cercopithecus mitis* **(G)**, *Gorilla gorilla* **(H)**, *Pan troglodytes* **(I)**, *Homo sapiens* **(J)**. See text for further information.

**Figure 9:**
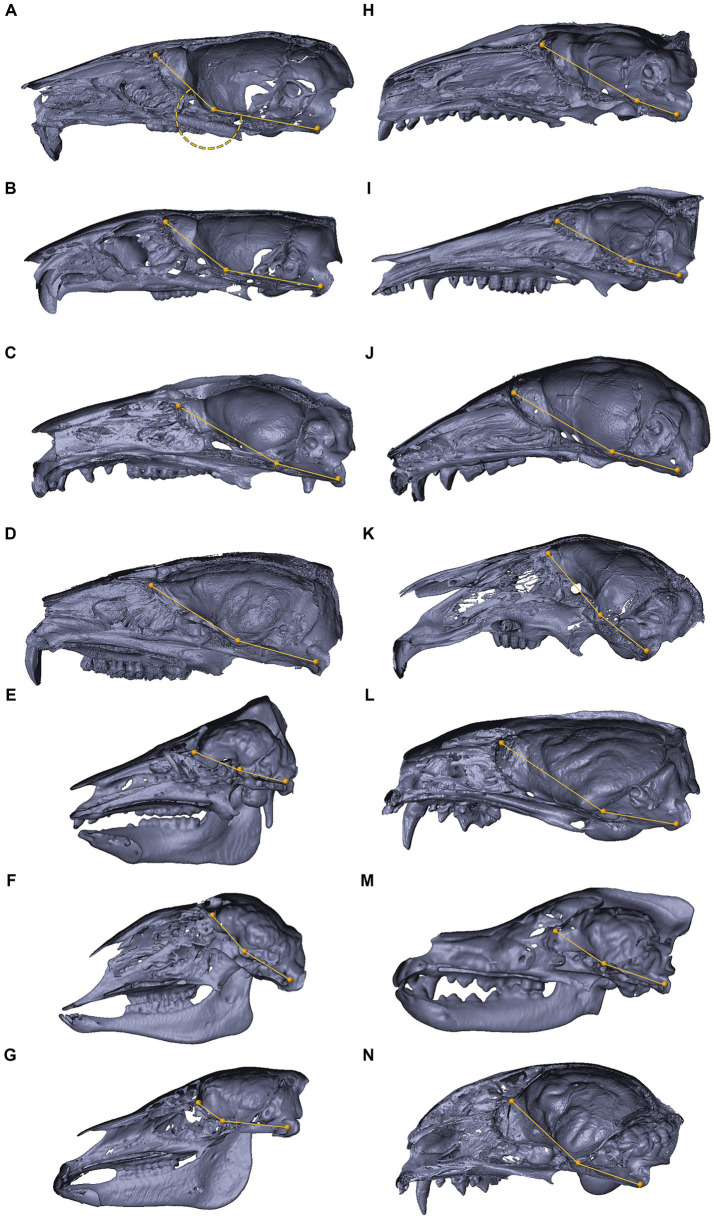
3D reconstructions of the cranial base in mammals. 3D reconstructions of mammalian crania sectioned along the midsagittal plane, representing inter-specific variations in the degree of cranial base flexion (CBA1). *Mus musculus* **(A)**, *Rattus rattus* **(B)**, *Trichosurus vulpecula* **(C)**, *Procavia capensis* **(D)**, *Sus scrofa* **(E)**, *Ovis aries* **(F)**, *Equus quagga* **(G)**, *Erinaceus concolor* **(H)**, *Isoodon macrourus* **(I)**, *Rousettus aegyptiacus* **(J)**, *Lepus capensis* **(K)**, *Vormela peregusna* **(L)**, *Canis lupus familiaris* **(M)**, *Felis silvestris catus* **(N)**. Refer to the text for landmarks details. Notably, for the examined species, the ventrally measured angle consistently exceeds 180°.

Figures 8, 9 depict 3D virtual reconstructions of primate and mammalian crania, respectively, based on their CT or μCT scans. The crania were sectioned along the midsagittal plane, and the landmarks defining the CBA1 were labeled. The CBA1 measurements are summarized in Table 2.

**Table 2:** Measurement of CBA1 for a single specimen from each species included in the present study.

Order	Species	CBA1 °
Primates	*Saguinus* sp. *Cholocebus pygerythrus* *Macaca fascicularis* *Saimiri* sp. *Cebus* sp. *Callithrix* sp. *Cercopithecus mitis* *Gorilla gorilla* *Pan troglodytes*	176.3 163.6 159.9 176.6 169.6 176.2 178.8 164.3 158.2
Rodents	*Mus musculus*	198.2
	*Rattus rattus*	194.7
Diprotodontia	*Trichosurus vulpecula*	197.8
Hyracoidea	*Procavia capensis*	197.1
Artiodactyla	*Sus scrofa*	185.5
	*Ovis aries*	194
	*Equus quagga*	210.5
Eulipotyphla	*Erinaceus concolor*	189.5
Peramelemorphia	*Isoodon macrourus*	190.2
Chiroptera	*Rousettus aegyptiacus*	193
Lagomorpha	*Lepus capensis*	188.9
Carnivora	*Vormela peregusna*	203.2
	*Canis lupus familiaris*	194
	*Felis silvestris catus*	200.9

**Figure 10:**
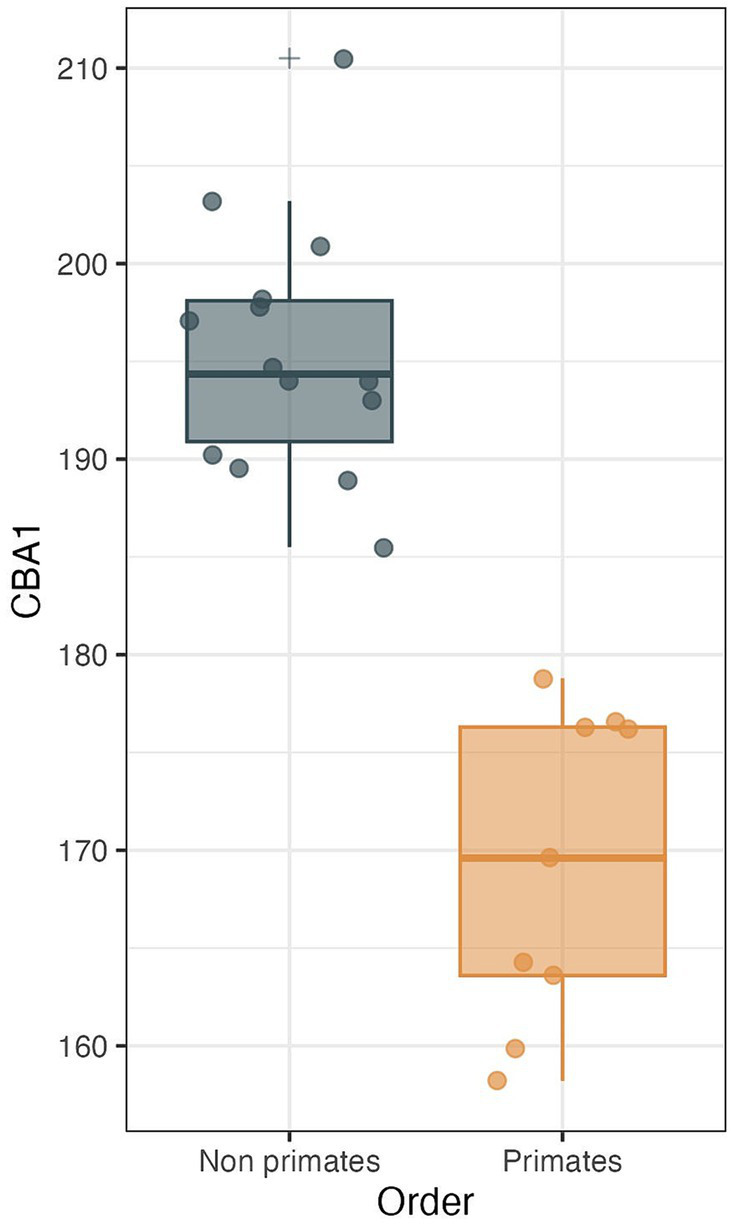
CBA1 measurements distribution boxplot comparing the distribution of CBA1 values between two groups: primates (*N* = 9) and non-primates (*N* = 14). The boxes represent the interquartile range (IQR), with the median depicted as a horizontal line inside each box. Whiskers extend to the minimum and maximum values within 1.5 times the IQR. Outliers are labeled using the ‘+’ marker symbol. The significant difference in CBA1 between groups is reflected in the *p*-value of 2.245e-08 obtained from a two-sample *t*-test.

Within the primate order, CBA1 measurements ranged between 158.2° and 178.8° with an average of 169.2° (*N* = 9) (Figure 8). None of the primate specimens we studied demonstrated a CBA1 larger than 180°. In all the mammalian species we examined, a consistent retroflexed orientation of the cranial base angle was observed, with CBA1 measurements ranging between 185.5° and 210.5° and averaging 195.5° (*N* = 14) (see Figure 10). In other words, the ventrally measured angle consistently surpassed 180°.

We conducted a two-sample t test to compare the means of two independent samples: primates (*N* = 9) and nonprimates (*N* = 14). The results indicate a *p* value less than the significance level of alpha = 0.05 (*t* = −8.6631, df = 21, *p* value = 2.245e-08). This suggests a significant difference between the average CBA1 of primates and nonprimates (*p* value <0.00001).

## 4 Discussion: an evolutionary perspective on abducens vulnerability

The abducens nerve plays a crucial role in ocular motility by innervating the ipsilateral lateral rectus muscle (Kiernan and Rajakumar, 2014; Kandel et al., 2021). Despite its functional significance, the abducens nerve is notably susceptible to injury in various clinical contexts, and understanding the anatomical basis for its vulnerability has long been a subject of interest in neurology and neurosurgery (Iaconetta et al., 2007; Tubbs et al., 2012). In this study, we employed a comparative neuroanatomical approach to investigate the course of the abducens nerve through the cranial base in various mammalian species, including primates, to gain a deep understanding of *why* the basicranial pathway of this nerve is unique in humans, predisposing it to injury in various clinical settings.

### 4.1 Comparative anatomy of the abducens nerve pathway

Our findings revealed consistent patterns in the course of the abducens nerve across different mammalian orders, with some notable anatomical variations in the gulfar segment. In humans and in nonhuman primates, the abducens nerve travels through Dorello’s canal, which is bounded by the petrous apex and the posterior clinoid process. Importantly, we observed several anatomical variations of the abducens nerve (e.g., duplication and pseudobranching), however in most cases the common single trunk variant was observed, in accordance with the available literature on abducens nerve variation (Nathan et al., 1974; Iwanaga et al., 2020; Wysiadecki et al., 2021b). This anatomical configuration poses a potential risk to the nerve due to tethering of its dural envelope to the dura mater covering the canal, as suggested by several authors (Umansky et al., 1992; Tubbs et al., 2012), and is present in the human cranial base from a very early stage of embryonic development (see Figures 4J, 6I). However, the degree of fusion between these bony and fibrous elements and the presence of an ossified or unossified petrosphenoidal ligament in the roof of Dorello’s canal can vary considerably between species.

In rodents, such as mice and rats, as well as other mammals examined in the present study, a distinct Dorello canal could not be identified. Instead, the abducens nerve was observed to pass beneath the pituitary gland, superior to the cavernous sinus, and then course along the lateral margin of the basisphenoid and presphenoid bones. These findings are in line with the few available descriptions of this region in rodents (Bleys et al., 1996). Artiodactyla and Perissodactyla species displayed variations in the location of the abducens nerve within the venous confluence formed by the petrous apex and the dorsum sella, further highlighting the complexity of the gulfar segment’s anatomy. These anatomical differences suggest that the susceptibility of the abducens nerve to injury in this segment may vary among species. Importantly, information regarding the vulnerability of the abducens nerve in nonhuman species is scarce. According to the available data, and in contrast to the situation in humans, the abducens nerve in these species is rarely injured in clinical contexts such as changes in intracranial pressure or trauma. Moreover, when affected, it is usually affected in conjunction with the oculomotor nerve, not in isolation (Penderis, 2003; Platt and Olby, 2014).

### 4.2 Why is the pathway of the abducens nerve unique compared to that of other cranial nerves?

In humans, the pathways of most cranial nerves are confined to a single cranial fossa, i.e., there is a short distance between their exit point from the brainstem and their respective cranial base foramen (Osborn et al., 2023). The olfactory bulb and tract are entirely situated within the anterior cranial fossa (López-Elizalde et al., 2018; Standring, 2021). The optic nerve emerges from the optic canal, passes through the optic chiasm above the pituitary gland, and immediately becomes embedded in the inferior surface of the hemisphere as the optic tract, and the situation is similar for the most caudal (lowest) cranial nerves (Rea, 2014; Standring, 2021). The facial and vestibulocochlear nerves emerge from the brainstem immediately in front of the internal auditory meatus, and their short pathway is thus confined to the posterior cranial fossa (Rea, 2014). The glossopharyngeal, vagus, and accessory nerves have a similar relation to the jugular foramen (Joo, 2024); the hypoglossal nerve, which exits from the medulla oblongata, has its own cranial base channel through the rim of the foramen magnum, the hypoglossal canal (Rhoton, 1979; Yang et al., 2021) with several types of variations (Öğüt et al., 2022).

In this respect, the pathways of the nerves that mediate ocular motility are unique because there is a longer distance between their exit point from the brainstem and the superior orbital fissure (Joo and Rhoton, 2015; Park et al., 2017). To explain this perspective on the abducens nerve pathway, we will first briefly discuss the pathways of the oculomotor, trochlear, and trigeminal nerves.

**Figure 11:**
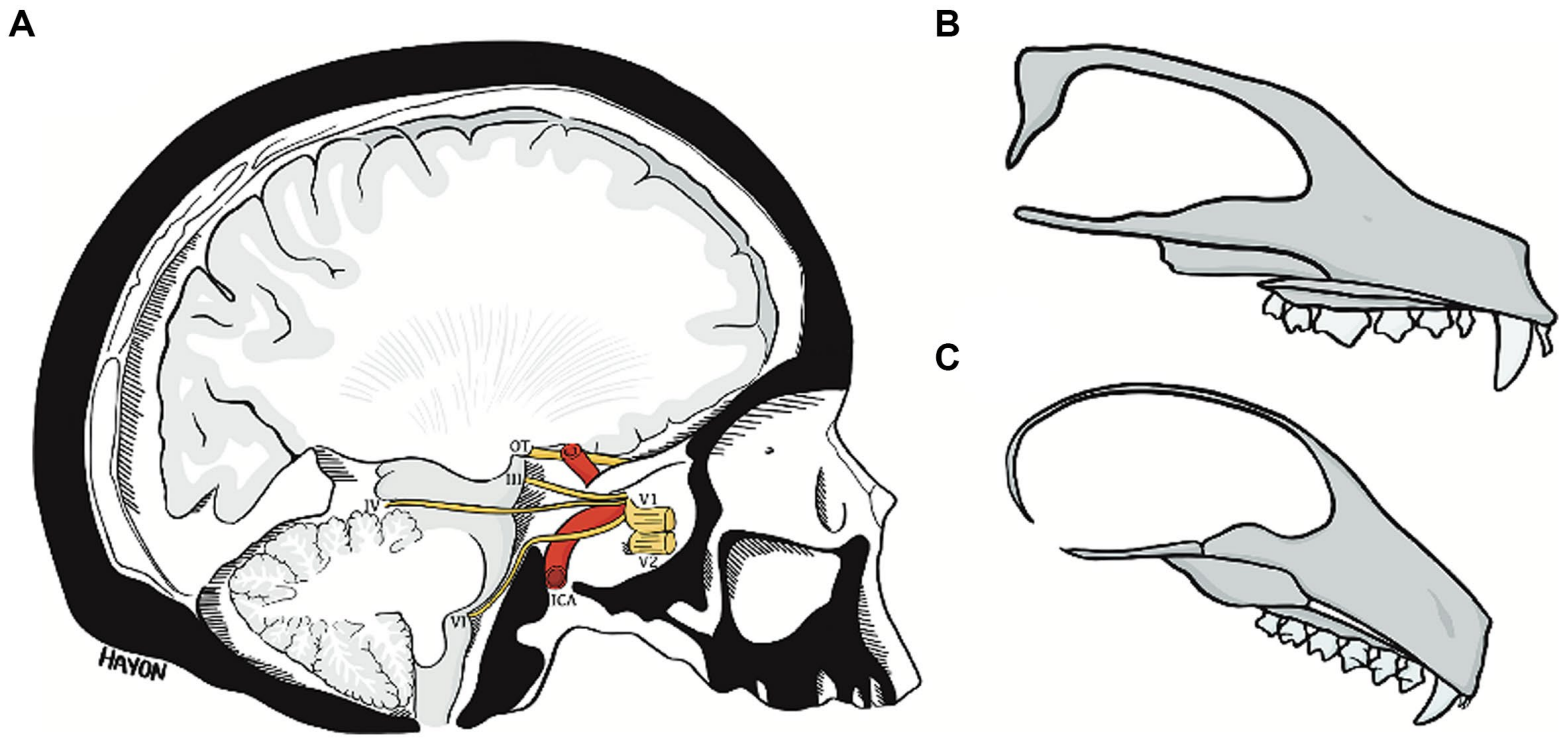
The abducens nerve pathway **(A)** an illustration of a parasagittal section through the human cranial base, modified from Wolff (1928). Note the relation of cranial nerves III, IV, and VI to the clivus and floor of the middle cranial fossa, and the marked angulation in the course of the abducens nerve. Compare the angulation of the human cranial base to the flat cranial base of a carnivore **(B)** and a lemur **(C)**. ICA, internal carotid artery; OT, optic tract; III, oculomotor nerve; IV, trochlear nerve; V1, ophthalmic nerve; V2, maxillary nerve; VI, abducens nerve.

The oculomotor and trochlear nerves exit the ventral and dorsal surfaces of the midbrain, respectively (Kiernan and Rajakumar, 2014). The pathways of these cranial nerves are included on a transverse plane, situated above the planes of other cranial nerves, such that they do not encounter any bony elements of the cranial base along their path (Tubbs and Oakes, 1998; Joo and Rhoton, 2015; Park et al., 2017; Standring, 2021). In addition, both nerves gain a special relation to the cavernous sinus, reaching it at the level of the free margin of the tentorium cerebelli and the posterior clinoid process (Meybodi et al., 2018). The meeting point with the tentorial margin introduces the oculomotor and trochlear nerves directly into the lateral wall of the cavernous sinus, which secures their course into the superior orbital fissure (Joo and Rhoton, 2015; Tubbs and Loukas, 2016; Park et al., 2017; Standring, 2021) (see Figures 3N, 11). Notably, trigeminal ganglion (of Gasser) is situated in Meckel’s cave (Standring, 2021), which establishes the connection between the prepontine cistern of the posterior cranial fossa and the cavernous sinus (Sicher and DuBrul, 1970; Rootman et al., 2022a). The ganglion itself lies on the surface of the middle cranial fossa immediately medial and anterior to the petrous apex, such that its ophthalmic and maxillary divisions are directly channeled into the lateral wall of the cavernous sinus, similar to the oculomotor and trochlear nerves, but inferior to them (Sicher and DuBrul, 1970; Joo et al., 2014) (see Figure 3N). These anatomical considerations are also demonstrated by our histological analyses of the lateral cavernous wall in human fetal specimens (see Figures 6A,F,G).

To conclude, the oculomotor, trochlear, and trigeminal nerves do not have to pass from the posterior to the middle cranial fossa by traversing the clivus or the petrous bone. This is because the oculomotor and trochlear nerves are above the clivus, and the trigeminal nerve is directed by Meckel’s cave immediately anterior to the clivus (Rea, 2014). This explanation is reminiscent of early attempts to explain the vulnerability of the abducens nerve (Collier, 1904).

The pathway of the abducens nerve is considerably more complicated. The abducens nerve exits the brainstem at the pontomedullary sulcus, much lower than the exit point of the oculomotor and trochlear nerves, which also travel to the superior orbital fissure (Sicher and DuBrul, 1970; Kandel et al., 2021). What distinguishes the abducens nerve pathway is that it bears a close relation to the bony walls of the posterior and middle cranial fossae (Sicher and DuBrul, 1970; Standring, 2021). Therefore, to reach the cavernous sinus, the abducens nerve must first ascend the clivus and then travel across the petrous bone; it is their presence in the highly flexed human cranial base that brings about the angulations in the course of the abducens nerve (Iaconetta et al., 2001, 2007). We contend that these constraints on the abducens nerve pathway underpin the emergence of the complex gulfar segment and the intricate relations of the nerve within Dorello’s canal and thereby its vulnerability.

### 4.3 Vulnerability of the human abducens nerve

The premise of the present study is that the situation described above for the human abducens nerve is markedly different in flat (unflexed) cranial bases, as demonstrated by our dissections of various mammals in the present study. In the flat cranial base, the exit point of the abducens nerve is simply more caudal – but not lower – than that of the oculomotor and trochlear nerves (Romer and Parsons, 1977). Accordingly, its pathway is straight and forward; namely, the pathway of the abducens nerve is not influenced by the morphology of the cranial base any more than that of other cranial nerves (Hill, 1935; Jones et al., 1983). These conclusions are also supported by our results regarding the spatial relations of the abducens nerve to the cavernous sinus. However, in humans, the abducens nerve passes through the sinus, and the other nerves in the region are embedded in the lateral wall of the sinus. In mammalian species, the relation of the abducens to the cavernous sinus is similar to that of the other regional nerves (compare plates C, D, H, I, and M in Figure 3). These observations are in accordance with the few available descriptions of the cavernous sinus in mammals (Bleys et al., 1996; Domingues et al., 1999).

In the context of the abducens nerve pathway, our study also provides some data regarding cranial base angulation in several mammalian species. In primitive mammals, the cranial base is a relatively flat plate at the floor of the endocranium (De Beer, 1937; Romer and Parsons, 1977). In higher mammals, especially humans, the planes at the base of the cranial fossae lie at angles relative to each other. Lieberman (2011) described the synchondroses between them as hinges between the cranial fossae at the midline. CBA1 is the most common metric of cranial base angulation and data regarding its measurements are mostly available for humans and primates (Lieberman, 2011). Adult humans have a significantly more flexed cranial base than other mammals, including primates (Lieberman et al., 2000; Lieberman, 2011). Our measurements of CBA1 in the mammals and primates we examined are aligned with these assertions. Although we measured CBA1 in a single specimen within each species, according to the results, in all the mammalian species, except primates, the cranial base is consistently retroflexed, with CBA1 measurements ranging between 185.5° and 210.5°. In contrast, in the primates we examined, CBA1 measurements ranged between 158.2° and 178.8°, and none of the primate specimens we studied demonstrated a CBA1 larger than 180°. This angle is of particular importance in our examination of the abducens nerve pathway, as this nerve emerges from the brainstem at a relatively low position and in close proximity to the midline. Furthermore, it also maintains close proximity to the cranial base floor throughout its course until it enters the cavernous sinus. Consequently, any structural changes in this floor, i.e., cranial base flexion, are directly reflected as alterations in the abducens nerve pathway. Taken together, these observations suggest that abducens nerve vulnerability in humans may be a price we pay for our highly flexed cranial base. As eloquently phrased by Richard Dawkins, “Nothing is free, everything comes with a price tag … Perfection in one department must be bought, in the form of sacrifice in another department … A body is a patchwork of compromises” (Dawkins, 2009; p. 70).

## 5 Limitations

Although the present study offers a novel perspective on the vulnerability of the abducens nerve, based on a comparative anatomical approach, it has several important limitations. First, only 10 mammalian orders, including primates, were included in this study. The mammalian class is highly diverse, such that the inclusion of more mammalian orders and more species within each order would strengthen the robustness of the results. Moreover, it would also allow a more accurate investigation of the interspecific variation. A second limitation concerns sample sizes. We examined 40 adult human hemicranial base specimens, 10 fetal human specimens, 18 primate specimens (of 9 primate species), and 18 mammalian specimens (of 9 mammalian species). Including more species and expanding the sample representing each species would not only enhance the significance of the results but also provide insight into intraspecific variation, which may play an important role in abducens nerve vulnerability. This limitation also applies to our measurements of CBA1 as an estimate of cranial base flexion. Last, the information available in the literature on abducens nerve vulnerability in primates and other mammalian species is scarce. More information on the vulnerability of this nerve in nonhuman species would significantly enhance our understanding of its susceptibility in humans.

## 6 Future directions

The present study aims to provide new insight into the vulnerability of the abducens nerve, through the application of a comparative neuroanatomical approach. An essential aspect of the abducens pathway is its vascular component, which deserves further exploration by delving into the cavernous segment of the abducens nerve. The dorsal meningeal artery, a branch of the meningohypophyseal trunk, is known to supply the petroclival portion of the abducens nerve and the proximal portions of the cranial nerves within the cavernous sinus (McCormack et al., 2021). Previous investigations have confirmed its intricate topographic relations and anatomical variability (McCormack et al., 2021), underscoring the importance of conducting a comparative analysis across nonhuman species. The importance of this research direction is highlighted by the variation observed in the carotid system and the cavernous sinus across mammals. For instance, the presence of the carotid rete mirabile, an arterial meshwork occurring at the cavernous portion of the internal carotid artery within the cavernous sinus, is notable in certain mammalian orders (e.g., artiodactyla) (Daniel et al., 1953). This variation highlights the need for comprehensive investigations to understand the implications of such anatomical differences on the vascular supply to cranial nerves, including the abducens nerve.

## 7 Conclusion

The comparative approach applied here suggests that cranial base flexion plays a critical role in the abducens nerve’s pathway and resulting vulnerability. Specifically, we suggest a distinction between cranial nerves that are almost entirely confined to one cranial fossa and those that span more than one cranial fossa. The olfactory and optic nerves are very close to their cranial base foramina, and the same is true for the caudal cranial nerves. In contrast, the oculomotor and trochlear nerves originate from the posterior cranial fossa and travel to the superior orbital fissure, traversing the middle cranial fossa. However, they are essentially situated above the clivus and the petrous bone. The trigeminal nerve reaches the middle cranial fossa immediately anterior to the petrous bone, i.e., it does not face the clivus or the posterior surface of the petrous bone. Collectively, the oculomotor, trochlear, and trigeminal nerves are channeled into their foramina by the tentorium and the lateral wall of the cavernous sinus. The only exception is the abducens nerve, which “experiences” the full extent of human basicranial flexion, as it faces both the clivus and the petrous bone of the posterior cranial fossa immediately after exiting the brainstem. Since its emergence from the pons is relatively medial, it does not attain a relation to the tentorium, and since its emergence point is relatively low, it cannot be channeled into the superior orbital fissure by the cavernous sinus wall.

This results in its complex passage from the posterior to the middle cranial fossa, its intricate anatomical relations, and notable vulnerability.

## Data availability statement

The original contributions presented in the study are included in the article/supplementary material, further inquiries can be directed to the corresponding author.

## Ethics statement

Given that the animal specimens utilized in the present study were obtained postmortem, Institutional Animal Care and Use Committee (IACUC) approval was not required, as confirmed in a letter from the Chair of our IACUC dated 1.6.2023. Human body donors included in the present study were obtained through our collaboration with Science Care (USA) for anatomical education and medical research, as evidenced by the Science Care Medical Certificate dated 6.3.2022. Additionally, approval for their use in research was granted by the Medical Directorate at the Ministry of Health (Approval no. 124473633123, 23.8.2023).

## Author contributions

LR: Conceptualization, Data curation, Formal analysis, Investigation, Methodology, Software, Supervision, Validation, Visualization, Writing – original draft, Writing – review & editing. AE: Conceptualization, Data curation, Investigation, Methodology, Project administration, Resources, Writing – review & editing. YS: Conceptualization, Data curation, Investigation, Methodology, Project administration, Resources, Writing – review & editing. RG: Methodology, Resources, Writing – review & editing, Formal analysis, Investigation. NE: Investigation, Methodology, Resources, Writing – review & editing, Conceptualization, Data curation. RC: Investigation, Methodology, Resources, Writing – review & editing. AM: Conceptualization, Data curation, Formal analysis, Funding acquisition, Investigation, Methodology, Project administration, Resources, Software, Supervision, Validation, Visualization, Writing – original draft, Writing – review & editing.
